# Single-cell RNA-sequence analysis of human bone marrow reveals new targets for isolation of skeletal stem cells using spherical nucleic acids

**DOI:** 10.1177/20417314231169375

**Published:** 2023-05-16

**Authors:** Elloise Z Matthews, Stuart Lanham, Kate White, Maria-Eleni Kyriazi, Konstantina Alexaki, Afaf H El-Sagheer, Tom Brown, Antonios G Kanaras, Jonathan J West, Ben D MacArthur, Patrick S Stumpf, Richard OC Oreffo

**Affiliations:** 1 Faculty of Medicine, Centre for Human Development, Stem Cells and Regeneration, Human Development and Health, Institute of Developmental Sciences, University of Southampton, Southampton, UK; 2 Cancer Sciences, Faculty of Medicine, University of Southampton, Southampton, UK; 3 College of Engineering and Technology, American University of the Middle East, Kuwait; 4 Physics and Astronomy, Faculty of Physical Sciences and Engineering, University of Southampton, Southampton, UK; 5 Department of Chemistry, Chemistry Research Laboratory, University of Oxford, Oxford, UK; 6 Chemistry Branch, Department of Science and Mathematics, Faculty of Petroleum and Mining Engineering, Suez University, Suez, Egypt; 7 Institute for Life Sciences, University of Southampton, Southampton, UK; 8 Mathematical Sciences, University of Southampton, Southampton, UK; 9 Joint Research Center for Computational Biomedicine, RWTH Aachen University, Aachen, Germany; 10 College of Biomedical Engineering, China Medical University, Taichung, Taiwan

**Keywords:** Stem cells, skeletal stem cells, nanoparticles, bone regeneration, single-cell RNA-sequencing

## Abstract

There is a wealth of data indicating human bone marrow contains skeletal stem cells (SSC) with the capacity for osteogenic, chondrogenic and adipogenic differentiation. However, current methods to isolate SSCs are restricted by the lack of a defined marker, limiting understanding of SSC fate, immunophenotype, function and clinical application. The current study applied single-cell RNA-sequencing to profile human adult bone marrow populations from 11 donors and identified novel targets for SSC enrichment. Spherical nucleic acids were used to detect these mRNA targets in SSCs. This methodology was able to rapidly isolate potential SSCs found at a frequency of <1 in 1,000,000 in human bone marrow, with the capacity for tri-lineage differentiation in vitro and ectopic bone formation in vivo. The current studies detail the development of a platform to advance SSC enrichment from human bone marrow, offering an invaluable resource for further SSC characterisation, with significant therapeutic impact therein.

## Introduction

The regenerative capacity of bone is essential for bone formation, remodelling and repair; evidence of the presence of an intrinsic skeletal stem cell (SSC) population. While this regenerative capacity has long been recognised, the in vivo identity of a skeletal stem cell population has only recently been confirmed.^1–3^ There is a wealth of data indicating human bone marrow derived stromal cells (HBMSCs) contain the SSC fraction with the potential to differentiate along the osteogenic, adipogenic and chondrogenic lineages.^2–6^ Skeletal progenitor populations have also been isolated from the growth plate and periosteum, demonstrating distinct multipotent and regenerative capabilities, but notably a lack of adipogenic potential in vivo.^1,7–9^

The availability of a SSC pool has garnered significant interest across the regenerative medicine community, given the potential clinical application.^10^ Despite this, current methods to isolate SSCs from human tissues remain challenging in the absence of a single specific marker for the SSC. The ability to isolate and study a homogenous SSC population would significantly advance understanding of SSC fate, immunophenotype, and simple selection criteria, all limiting factors in the widespread clinical application of these cells. Although, a range of cell surface markers can enrich for SSCs, there remains a lack of consensus within the field and relevant literature, and none of the proposed markers, alone, holds the potential to identify and isolate a homogenous SSC population.^1,8,11,12^ Furthermore, given the heterogeneity of SSC populations derived from different sources,^13,14^ there remains a need to better characterise the SSC identity to reveal novel markers that characterise SSCs with regenerative functionality in vivo.

Cell fate in culture, tissues, and organisms can be followed using a variety of techniques, although cell phenotype information is typically limited to cell surface epitopes.^15^ The detection of a specific mRNA responsible for the expression of a certain protein can be used to determine and characterise the phenotype of the cell. However, traditional methods to assess specific mRNAs such as in-situ hybridisation, northern blot, or quantitative-PCR necessitate cell fixation or lysis to isolate the RNA, resulting in loss of the cells for further experiments.

In recent years, DNA-coated spherical nanoparticles, also termed spherical nucleic acids (SNAs), have emerged as novel nanomaterials within the biomedical field.^16–18^ The three-dimensional packed arrangement of oligonucleotides around a spherical nanoparticle core, endows SNAs with unique properties including efficient cellular uptake in the absence of a transfection agent, increased endocellular stability in the presence of nucleases as well as enhanced selectivity and specificity towards their complementary sequence.^17,19^ As a result, SNAs have been used as tools for the regulation of gene expression, drug delivery as well as the detection of RNA targets including microRNA and mRNA.^16,20–28^ Recently, we have demonstrated how mRNA targets can be imaged in the live cell in real time both in vitro and in vivo.^29,30^ Furthermore, we have shown the development of SNAs with dual functionality capable of delivering a drug payload upon the detection of a specific mRNA target.^31,32^ The intrinsic properties of SNAs have therefore paved the way towards their use in relevant applications.

Here, we demonstrate how a single-cell RNA sequencing (scRNA-seq) platform, Drop-seq, can be used to identify candidate markers of human stem and progenitor skeletal populations. The identified markers serve as SNA targets for rapid isolation of viable human cells, using mRNA signatures detected in skeletal cells, in real time. Drop-seq allows full transcriptome sequencing of upwards of tens of thousands of individual cells within a single experiment.^33^ We have used Drop-seq to generate mono-disperse nanolitre-sized droplets at >2 kHz for single cell capture. These droplets are stochastically loaded with single cells and, following cell lysis, poly-adenylated transcripts from individual cells are captured on functionalised micro-particles. Gene-of-origin and cell-of-origin are later reconstructed using the combined sequence information of transcript and the DNA barcode specific to each micro-particle. The current work demonstrates how Drop-seq can be utilised to uncover the transcriptomic signatures of bone marrow subpopulations, and to subsequently identify transcripts that distinguish SSCs from other cell types. ScRNA-seq analysis revealed candidate markers, including Hevin (*SPARCL1*) and Transferrin (*TF*), to serve as SNA targets to select cells expressing desired mRNA signatures. The enriched SSC populations were validated through in vitro clonogenic and differentiation assessment, and in vivo heterotopic bone formation. The current approach provides new targets and a platform to advance human bone marrow SSC isolation and enrichment with significant therapeutic impact.

## Results

### ScRNA-seq of human adult bone marrow reveals SPARCL1 as a candidate marker of skeletal progenitors

**Figure 1.**
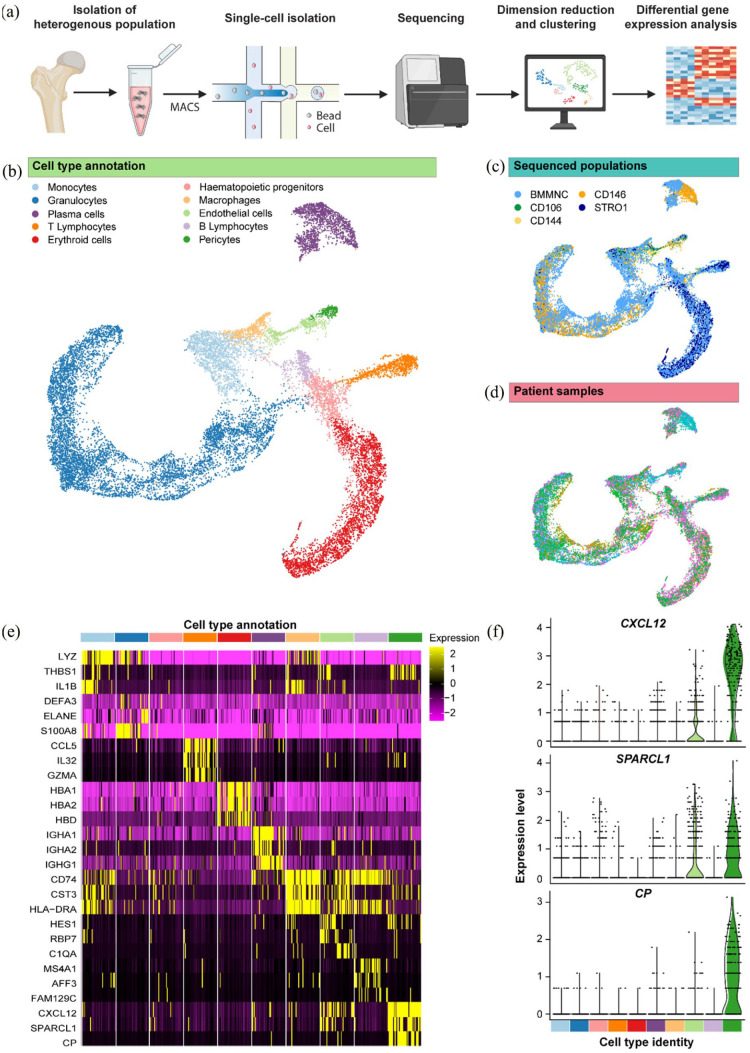
ScRNA-seq of 17,102 human adult bone marrow cells reveals SPARCL1 as a candidate marker of skeletal progenitor populations. (a) Experiment overview. Heterogeneous bone marrow populations were isolated from human adult bone marrow and MACS was applied to enrich for a CD45−/CD146+ skeletal progenitor population, CD144+ endothelial cells and CD144−/CD106+ pericytes. Single cells were isolated and sequenced following the Drop-seq methodology. Unsupervised clustering was applied to reveal subpopulations, which were characterised by differential gene expression to identify lineage biomarkers. (b) Uniform Manifold Approximation and Projection (UMAP) was performed to reduce the dimensionality of, and visualise, the normalised gene expression, each point representative of a single cell. Cluster analysis revealed eight distinct groups corresponding to haematopoietic and non-haematopoietic cell types. (c) The sequenced population and (d) patient sample plots show the integration of data from distinct sources across the UMAP. (e) Heatmap of normalised expression of the top three differentially expressed genes from each cell type cluster in comparison to all other cells. Each cluster was down-sampled to 200 cells. (f) Violin plots of expression of top three most significant differentially expressed genes in the pericyte cluster; CXCL12, SPARCL1 and CP (for all targets, significance <0.001).

The in vivo identity and origin of the SSC remains highly elusive, and while several recent high-impact studies have focused on characterisation of growth plate resident SSCs,^9^ a sub-endothelial perivascular origin of the SSCs in bone marrow is widely acknowledged.^2,34–36^ We applied scRNA-seq, using the Drop-seq methodology,^33^ to characterise the cellular heterogeneity within human adult bone marrow to identify potential markers for enrichment of bone marrow-derived SSCs (Figure 1(a)). We profiled 9795 cells from freshly isolated human bone marrow from three individuals undergoing hip replacement surgery. To enrich the pool of perivascular cells for scRNA-seq analysis, patient samples were fractioned into three cell populations; the previously described CD45−/CD146+ skeletal progenitor population,^2^ CD144+ endothelial cells,^37^ and CD144−/CD106+ pericytes.^38^

The sequenced libraries were combined with a previously published scRNA-seq dataset consisting of unfractionated human bone marrow populations and Stro-1+ enriched populations from three patients^39^(data available from ArrayExpress under accession number E-MTAB-8630), enabling comparison between depleted and unfractionated bone marrow. After quality filtering, the total integrated data, comprised 17,102 cells with an average of 2097 transcripts per cell (dataset referred to as Drop-seq1). An unsupervised clustering strategy was employed to detect distinct cell subtypes with unique gene expression signatures.^40^ The default cluster analysis method implemented in Seurat (Louvain clustering,^41^), takes into account the normalised gene expression values of all cells and groups them based on the similarity of their gene expression profiles. Using default parameters, 10 cell clusters were identified, corresponding to haematopoietic and non-haematopoietic cell types. Since gene expression profiles obtained from scRNA-seq contain thousands of genes, they are difficult to visualise. To address this problem, a technique called UMAP was used to project the high-dimensional expression vector into two dimensions, where cells can be visualised in a scatter plot (Figure 1(b)). In this representation (Figure 1(b)), the colour-coding indicates the group to which a cell was assigned. Notably, clustering and 2D projection corroborate the finding that cells of similar gene expression signature are grouped in a robust and biologically meaningful way (Figure 1(b)). To demonstrate that clustering and proximity are due to expression differences of distinct genes, expression levels of individual genes were superimposed onto the 2D projection (see Figure S1). These results support the biological interpretation that clusters correspond to cell types, which can be identified by the expression of unique marker genes. Guided by the localised expression of established cell type markers, clusters were broadly labelled as haematopoietic progenitors (*CD34*+, *SPINK2*+), granulocytes (MPO+,LTF+), erythroid cells (*HBD*+, *CA1*+), B lymphocytes (*MS4A1*+, *BANK1*+), plasma cells (*MZB1*+, *IGKC*+), monocytes (*CD14*+, *VCAN*+), macrophages (*FGL2*+, *HLA*-*DRA*+), T lymphocytes (*CD8A*+, *IL7R*+), endothelial cells (VWF+, CLEC14A+) and pericytes (*LEPR*+, *CXCL12*+) (Figure S1). Visualisation of sequenced populations and patient samples following integration of the data confirmed clustering was not due to differences in samples/batch effects (Figure 1(c) and (d)). Interestingly, despite enrichment of perivascular subtypes prior to sequencing, all cell types were represented within the sequenced populations (Figure S2).

Differential gene expression analysis was performed on the raw data to compare the molecular signature of each cluster with all other cells (Figure 1(e)). To identify candidate SSC markers, we examined the transcriptomic pattern within the pericyte cluster, comprising 222 cells. Analysis revealed several potential genes of interest, not previously fully explored within the context of SSC enrichment, among which, C-X-C Motif Chemokine Ligand 12 (*CXCL12*), SPARC Like 1 (*SPARCL1*) and Ceruloplasmin (CP) were the three most differentially expressed genes in pericytes (Figure 1(f)). Expression of these mRNA markers was highly limited to perivascular cell types; expressed in 91%, 67% and 69% of pericytes, respectively. SNAs were designed against *CXCL12, SPARCL1* and CP to evaluate the enrichment of SSCs following isolation targetting these mRNA sequences (Figure S3: schema of mRNA detection using SNAs).

### SNAs targetting SPARCL1, SOST, CD200 and CD146 mRNA enrich for CFU-F

**Figure 2.**
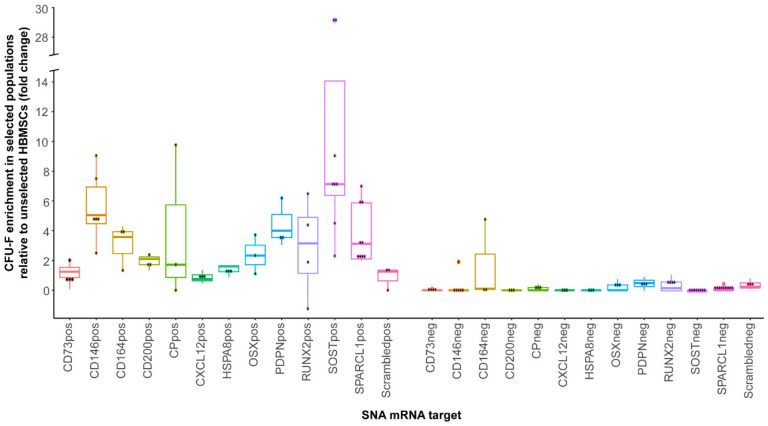
Cells enriched for CD146, CD200, SOST and SPARCL1 targets demonstrate enhanced CFU-F capacity in comparison to unselected cells. SNAs were designed to target a wide range of molecular markers, identified from scRNA-Seq libraries and related literature. The positive and negative populations were plated at 5000 cells per well of a 12-well plate and colonies were counted after 2 weeks of culture. For each SNA target, each point represents the mean CFU-F count from a different patient plated as triplicates, displayed as a percentage of unsorted CFU-F counts. Each SNA target was assessed in a minimum of three patients.

To test suitable candidate SNAs for SSC isolation, SNAs were designed targetting a range of mRNAs; these included *SPARCL1*, *CXCL12* and *CP*, as identified by scRNA-seq, and other SNAs including: *HSPA8* (encodes antigen detected by STRO-1 antibody),^42^*RUNX2*,^43^ and a scramble sequence that did not detect any target mRNA. Other potential mRNA targets were *CD164, CD146*,^2^*SP7* (*Osterix*)^43^ and Sclerostin (*SOST*).^44^ Recently, Chan et al. 2018 and Debnath et al. 2018 have indicated likely candidate protein markers for SSCs,^1,8^ and thus, SNAs were designed targetting *CD73*, *CD200* and *PODOPLANIN* (*PDPN*). Following incubation with the SNAs, positive (bright) and negative (dim) fractions were collected (see Methods). Each SNA was tested against a minimum of three different patients, and up to three SNAs were tested for each patient. In total, bone marrow samples from 18 different patients were tested. To determine the clonogenic capacity of SNA-sorted populations, cells were plated at limiting dilution, whereby each fibroblastic colony formed, is derived from a single SSC or progenitor, termed CFU-F.^45^ The normalised CFU-F counts as a percentage of the unsorted cells are presented in Figure 2. Enrichment of CFU-F in comparison to scrambled control and unsorted cells was observed in all positive fractions, while the negative fractions displayed minimal CFU-F.

SNAs displaying the most consistent CFU-F enrichment, and specificity for the positive cell fraction in comparison to unsorted cells, were *CD200* (1.85), *SPARCL1* (3.76), *CD146* (5.58), *NANOG* (3.96), *SOST* (11.93) and *OSTERIX* (2.39) (values in brackets depict mean fold-change of CFU-F enrichment in selected populations relative to unselected HBMSCs). Interestingly, the *OSTERIX* SNA also demonstrated regular CFU-F enrichment in the negative cell fraction (0.24). In agreement with the scRNA-seq data, *SPARCL1* mRNA facilitated sorting of cells with enhanced clonogenic functionality. The highest levels of CFU-F enrichment were observed in SOST+ populations, presenting *SOST* as a candidate SSC biomarker. Similarly, *CD200*+ and *CD146*+ were defined as populations of interest for further scRNA-seq analysis. To evaluate the capacity of these targets to isolate multipotent SSCs, we enriched *SOST*+, *SPARCL1*+, *CD146*+ and CD200+ cell populations and determined the osteogenic, chondrogenic and adipogenic potential in vitro (Figure S4). We found that *SOST*+, *CD146*+ and SPARCL1+ cells possessed the capacity for tri-lineage differentiation, confirmed through histological assays (Figure S4). We were unable to obtain sufficient cell numbers to assay CD200+ populations. Elevated CFU-F enrichment was also observed in NANOG+ population (Figure S5), in accordance with the role of *NANOG* in stem cell maintenance,^46,47^ however, we were unable to collect enough NANOG+ cells for any downstream analysis.

### ScRNA-seq reveals TF, DCN, CALD1, COL1A2 and FMO3 as candidate targets for SSC enrichment

**Figure 3.**
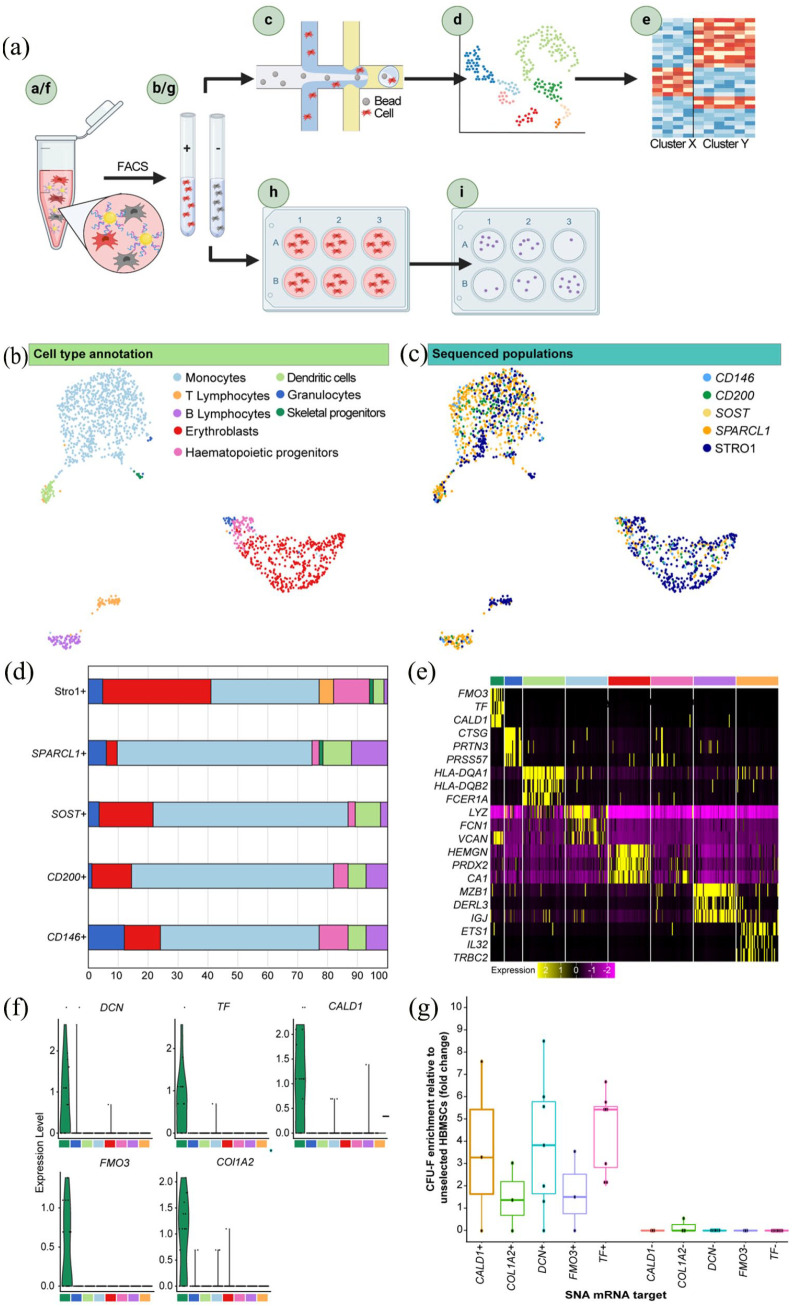
ScRNA-seq analysis of 1573 enriched populations identified candidate markers for SSC isolation. (A) Experiment overview. (a) Heterogeneous bone marrow populations were isolated from human adult bone marrow and incubated with SNAs targetting CD146, CD200, SOST and SPARCL1. (b) FACS was performed, and positive fractions were collected based on SNA fluorescence. (c) Enriched populations, together with a MACS Stro-1+ populations, were sequenced, using the Drop-seq methodology. (d) Clustering analysis revealed a cluster of skeletal progenitor cells. (e) Differential gene expression analysis identified candidate skeletal progenitor biomarkers. (f) SNAs were designed to target the candidate biomarkers and incubated with heterogenous bone marrow. (g) As before, FACS was performed, and positive fractions were collected based on SNA fluorescence. (h) Enriched cell populations were plated at limited dilution and incubated in basal medium. (i) After 2 weeks, cell colonies were stained with crystal violet to determine CFU-F enrichment relative to unselected bone marrow populations. (B) UMAP was performed to reduce the dimensionality of, and visualise, the normalised gene expression, each point representative of a single cell. Unsupervised clustering identified eight distinct cell types of haematopoietic and non-haematopoietic lineages. (C) Visualisation of sequenced populations on the UMAP plot show integration of samples. (D) Populations were randomly down-sampled to equal numbers (83 cells/population) and the cluster type of each sequenced population was determined, and revealed the skeletal progenitor cluster comprised SPARCL1+ and Stro1+ cells only. (E) Heatmap of normalised expression of the top three differentially expressed genes from each cell type cluster in comparison to all other cells. Cells in each cluster were down-sampled to 50 cells. (F) Violin plots of expression of top five most differentially expressed genes in the skeletal progenitor cluster; FOM3, TF, DCN, CALD1 and COL1A2. (G) SNAs were designed to the DCN, TF, CALD1, COL1A2 and FMO3 mRNA. The positive and negative populations, together with unsorted cells, were plated at 5000 cells per well of a 12-well plate and colonies were counted after 2 weeks of culture. For each SNA target, each point represents the mean CFU-F count from a different patient plated as triplicates, displayed as a percentage of unsorted CFU-F counts. Each SNA target was assessed in a minimum of three patients. In total, bone marrow samples from 17 different patients were tested. Horizontal bars represent median values.

In order to increase the SSC pool for scRNA-seq, Drop-seq of MACS-collected Stro-1+ cells, and cells enriched using SNAs targetting *CD146, CD200*, *SOST* and *SPARCL1* mRNAs was performed (Figure 3(a)). In total the transcriptomes of 1900 cells were sequenced, producing a dataset of 1573 cells with an average of 1899 transcripts per cell following quality control filtering. Unsupervised clustering identified eight distinct cell types: Skeletal stem cells/progenitors, monocytes, erythroblasts, granulocytes, B lymphocytes, dendritic cells, T lymphocytes and haematopoietic stem cells (Figure 3(b)). Cluster identity was assigned by expression of lineage biomarkers (Figure S6). The smallest cluster, comprising 15 cells, was annotated as ‘Skeletal Progenitors’ based on expression of stromal markers *CXCL12*^48^ and *LEPR*,^4^ stem cell factor *KITLG*, and the expression of *SPARCL1* and *CD200*; shown to enrich CFU-F following SNA-based cell sorting.

All sequenced populations were observed to be heterogenous (Figure 3(c)). To evaluate the cluster type composition of each population, samples were down-sampled to equal cell numbers (83 cells) and the proportion of cells annotated in each cluster was quantified (Figure 3(d)). The skeletal progenitor cluster represented 0.63% and 1.88% of *SPARCL1* and STRO-1 populations, respectively. Overall, the sequenced populations maintained a degree of heterogeneity and included cells across the classified cell-types; SPARCL1+ cells were represented in all eight cell-type subpopulations (Figure 3(d)). Despite this observed heterogeneity, the SNA methodology was found to enrich for monocytes; with *CD146*+, *CD200*+, *SOST*+ and SPARCL1+ populations consisting of 56%, 42%, 68% and 66% monocytes, respectively (Figure 3(d)); reflective of the FACS gating strategy, described previously.^49^ The Stro1+ cells were largely erythroblasts (51%), consistent with the findings of Simmons and Torok-Strob who demonstrated that CFU-F predominantly resided within the Stro-1+/GYPA- fraction.^50^ Differential gene expression analysis was performed to characterise the molecular signature of the skeletal progenitor cluster in comparison to all other cell types (Figure 3(e)). The five most differentially expressed genes were *TF*, Flavin-containing monoxygenase-3 (*FMO3*), Decorin (*DCN*), Caldesmon (*CALD1*) and Collagen Type I Alpha 2 Chain (*COL1A2*) (for all targets the Wilcoxon Rank Sum Test determined significance, *p* < 0.001) (Figure 3(f)). The highest average log2 fold-change score was calculated for *TF*; 1.55 Avg_Log2FC with *TF* expressed by 66.70% of cells within the skeletal progenitor cluster, and less than 0.10% of other cells within the dataset. Furthermore, *TF* was shown to have elevated expression in the pericyte cluster identified in the initial scRNA-seq dataset (Drop-seq1) (Figure S7).

To further validate the specificity of the candidate SSC markers, identified by scRNA-seq, we used publicly available bone marrow scRNA-seq data, to evaluate the expression of *TF, DCN, CALD1, COL1A2, FMO3* and *SPARCL1* (Figure S8-S10). Using an interactive web portal to view the distribution of target gene expression across haematopoietic and non-haematopoietic populations in the human cell atlas bone marrow data,^51^ we observed elevated expression of candidate markers in the stromal populations, with median expression values of 3.76 (*TF*), 3.63 (*DCN*), 3.14 (*CALD1*), 2.20 (*COL1A2*), 2.13(*FMO3*) and 1.57(*SPARCL1*) (Figure S8).

We also performed analysis of scRNA-seq data generated by Wang et al. and colleagues, comprising >14,000 CD271^+^ bone marrow mononuclear cells (BM-MNCs) obtained from two individual donors.^52^ Within the heterogeneous CD271^+^ BM-MNCs, Wang et al. identified a cluster of *LEPR*^high^*CD45*^low^ cells which were annotated as bone marrow mesenchymal stem cells (BM-MSCs), which we refer to herein as SSCs (Figure S9A). Interestingly, this cluster was also shown to express several other SSC markers; LEPR^+^CD73^+^CD105^+^CD90^+^ (Figure S9B). We mapped expression of the key markers we identified through scRNA-seq of MACS and SNA-selected cell populations onto the Wang et al. dataset and observed elevated expression of *TF, DCN*, *CALD1, COL1A2, FMO3* and *SPARCL1* in the SSC cluster (Figure S9C). As performed by Wang et al. we extracted the *LEPR*^high^*CD45*^low^ cells to identify subpopulations within the SSC cluster (osteogenic progenitors, adipogenic progenitors, terminal cells and contaminate cells)^52^ (Figure S9D). Expression of *TF*, *DCN*, *SPARCL1*, *CALD1*, *COL1A2* and *FMO3* were shown to be expressed by all SSC subpopulations, suggesting these markers do not show commitment to a specific SSC lineage (Figure S9E-F).

Finally, for completeness, although cognisant of the species difference, we assessed expression of our selected markers across Dolgalev and Tikhonova’s integration of mouse bone marrow niche datasets,^53^ comprising 32,743 cells across five recent studies.^54–58^ We found the expression of *Trf, Dcn, Cald1, Col1a2, Sparcl1*, and *Fmo3* to be less comparable to expression of the ortholog genes in human bone marrow datasets (Figure S10). For example, *Sparcl1* was expressed in fibroblasts and arteriar and arteriolar endothelial cells, but lowly expressed in cells annotated as mesenchymal progenitor cells (MSPCs) (Figure S10B), and *Fmo3* showed very low expression across all cell types in the mouse bone marrow data (Figure S10C). Consistent with our scRNA-seq analysis in human bone marrow, *Trf* was differentially expressed in MSPCs of osteogenic and adipogenic lineages, in comparison to all other cell types. However, Trf was also expressed by endothelial sinusoidal cells (Figure S10D).

In addition to validating the differential expression of the target markers in skeletal progenitor populations, we assessed the functionality of the novel markers, for isolation of SSCs. We designed SNAs targetting *TF*, *FMO3*, *DCN*, *CALD1* and *COL1A2*, and incubated HBMSCs with the SNAs for 1 h, prior to collecting positive and negative Cy5 cell fractions. SNAs were tested on a minimum of three different patient samples per target. For each sample, positive, negative, and unsorted cells were plated at 5000 cells per well of a 12-well plate to assess SSC enrichment within each fraction. Following crystal violet staining, visible clusters were counted, indicative of the number of CFU-F enrichment. The number of CFU-F for each selected population was expressed as a percentage of unsorted cells (Figure 3(g)). For each SNA-target, enrichment for CFU-F was observed in all positive fractions relative to unselected HBMSCs. Additionally, no enrichment of CFU-F was observed in negative fractions. TF+ populations demonstrated the most consistent elevation of CFU-F (4.5 average fold change relative to unselected HBMSCs) and was found to enhance colony formation in all patient samples assessed. Although the highest enrichment was observed in DCN+ population (8.7 average fold change), one DCN+ population did not enrich CFU-F relative to unselected HBMSCs.

MACS provides a less time-consuming approach to cell sorting of large cell numbers than the SNA-based FACS strategy applied in this study, although MACS relies on the detection of surface epitopes.^59^ It was therefore of interest to investigate whether the same level of SSC enrichment, observed following SNA-based cell sorting, could be achieved using MACS, with DCN and TF as antigen targets. Negative and positive fractions were collected for three samples and cells were plated at 50,000 cells per 3 wells of a 12-well plate for CFU-F assessment. In marked contrast to the CFU-F enrichment observed in DCN+ and TF+ populations following SNA mRNA detection, cells sorted using DCN and TF antibodies did not display CFU-F capacity in positive fractions following MACS (Figure S11).

### Incubation with two SNAs targetting different mRNAs collects cell populations with the capacity for CFU-F enrichment and tri-lineage differentiation

Following the identification of SNA targets which enrich populations with enhanced CFU-F capacity, indicative of SSC enrichment, we performed further studies to evaluate whether functionality could be enhanced by targetting different mRNAs simultaneously. *SPARCL1*+ was found to enrich comparable numbers of CFU-F to the classical SSC enrichment method using MACS to select Stro-1+ cells (Figure S12).^60^ Furthermore, STRO1+SPARCL1+ cell populations displayed a significantly enhanced clonogenic capacity than Stro1+ cells alone (*p* < 0.05) (Figure S12).

**Figure 4.**
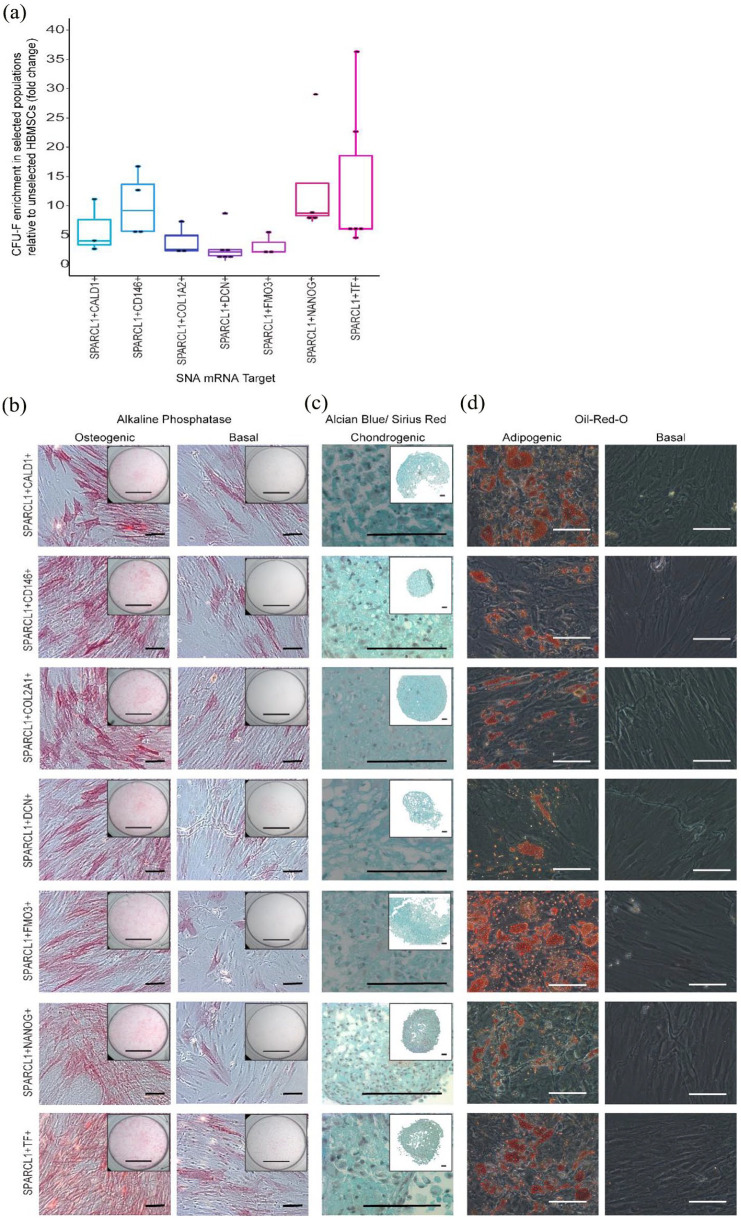
Cell populations selected based on dual expression of two mRNA targets display enhanced levels of CFU-F and capacity for tri-lineage differentiation. (a) CFU-F following dual SNA-selection. Cells were incubated with SPARCL1 Joe-tagged SNAs, and Cy-5 tagged SNAs targetting CD146, CALD1, DCN, COL1A2, FMO3, NANOG, or TF. The positive and negative fluorescent cells were collected and plated for CFU-F. For each SNA combination, each point represents the mean CFU-F count from a different patient, displayed as a percentage of unsorted CFU-F counts. In total, bone marrow samples from 18 patients were tested. Horizontal bars represent median CFU-F values. To assess tri-lineage capacity, cell populations were collected and expanded in vitro. (b) Osteoinduction. Cells were cultured in basal medium with 50 µM ascorbic acid 2-phosphate and 10 nM vitamin D3 for 14 days. Mineralisation is shown using alkaline phosphatase staining. Scale bars = 100 µm, whole well = 500 µm. *n* = 3. (c) Chondrogenic induction. Cells were cultured in basal medium supplemented with 100 µM ascorbic acid 2-phosphate, 10 ng/mL TGF-B3, 10 µg/mL ITS solution, 10 nM dexamethasone. Alcian blue/Sirius Red staining revealed proteoglycan synthesis (blue denotes proteoglycan deposition, purple indicates collagen deposition). Scale bars = 100 µm. *n* = 3. (d) Adipogenic induction. Cells were cultured in basal medium with 100 nM dexamethasone, 500 µM IBMX, 3 µg/mL ITS solution, and 1 µM rosiglitazone for 14 days. Oil-Red-O staining indicates lipid droplet formation. Scale bars = 100 µm. Results for each target were obtained from three different patient samples: representative images are shown.

Based on these findings, we designed a second generation SNA, targetting *SPARCL1*, using JOE fluorescent dye in the place of Cy5 on the 5′ end of flare strands. Changing the dye on the *SPARCL1* SNA flare strand prevented confusion of the signal detected when using two SNAs in combination, enabling us to collect cells expressing both *SPARCL1* mRNA and a second target of interest. The choice of dye made no difference to the CFU-F count isolated (data not shown). Cells were incubated with JOE labelled *SPARCL1* SNAs, together with Cy5 labelled SNAs targetting either *CD146*, *CALD1, COL1A2, DCN, FMO3, NANOG*, or *TF.* The top 10% Cy5+/JOE+ fluorescent cells were collected and plated for CFU-F assessment. SPARCL1+TF+, SPARCL1+NANOG+ and SPARCL1+CD146+ cells demonstrated the most enhanced CFU-F enrichment compared to unsorted cells (Figure 4(a)). To assess the stem cell potential of these enriched subpopulations, SNA-selected cells were expanded in vitro and cells were cultured under conditions favourable for induction of SSC tri-lineage differentiation, an established criteria defining SSCs (Figure 4(b)).^61^ For osteoinduction, cells were cultured in basal medium with 50 µM ascorbic acid 2-phosphate, and 10 nM vitamin D3 for 14 days. Histological analysis revealed enhanced expression of alkaline phosphatase, indicative of osteogenic differentiation, in SNA-sorted populations following culture in osteoinductive media in comparison to basal cultured populations (Figure 4(b)). For chondrogenic differentiation assessment, cells were cultured as pellets in basal medium with 100 µM ascorbic acid 2-phosphate, 10 ng/mL TGF-B3, 10 µg/mL ITS solution, 10 nM dexamethasone. Alcian blue staining of the chondrogenic pellets revealed proteoglycan synthesis in all SNA-sorted populations. However, Sirius Red stain retention was absent in all pellets, except minimal staining observed in SPARCL1+NANOG+ cells, indicating no collagenous matrix formation. To evaluate the adipogenic potential of the SNA-sorted populations, cells were cultured in 100 nM dexamethasone, 500 µM IBMX, 3 µg/mL ITS solution, and 1 µM rosiglitazone. Oil-Red-O staining of lipids was performed after 14 days of culture, evidencing induction of adipogenesis and lipid droplet formation, with enhanced levels of lipid droplets observed in SPARCL1+NANOG+ and SPARCL1+TF+ populations (Figure 4(d)). In summary, we evidenced the enhanced proliferation capacity and multi-lineage potential of SNA-sorted populations following culture in supplemented media, supporting the classification of the cells as enriched SSC populations.

### TF+ cells produce mineralised tissue in vivo

**Figure 5.**
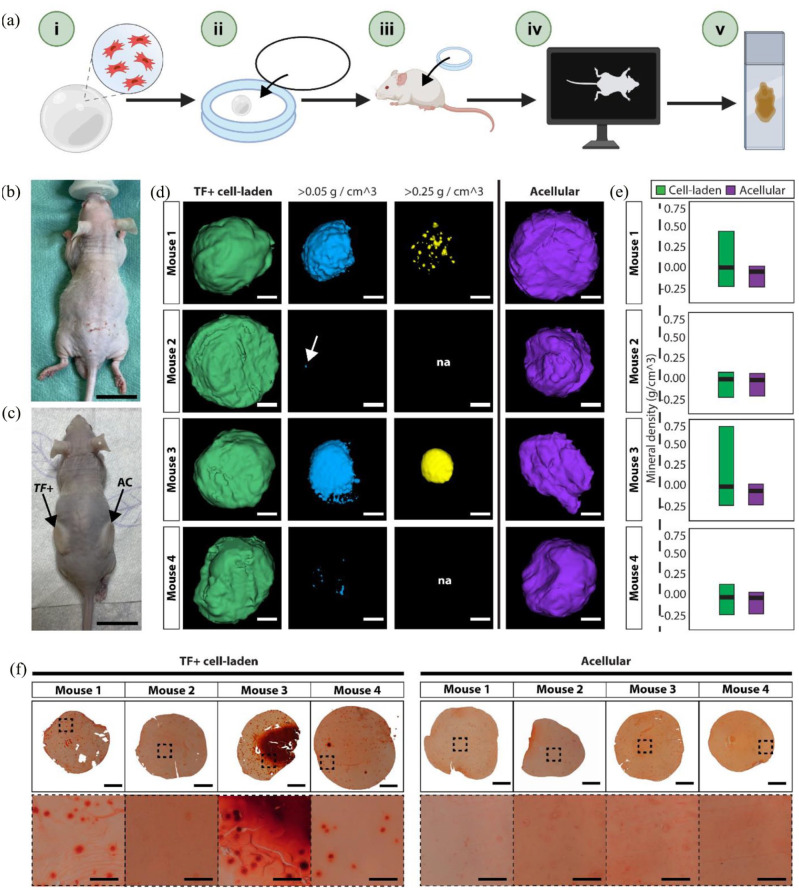
TF+ cell-laden scaffolds form mineralised nodules after 8-weeks in vivo. A total of four mice were used to evaluate bone formation in TF+ populations. (a) Overview of the in vivo methodology: (i) enriched TF+ cells were encapsulated in alginate/chitosan polysaccharide capsules at a concentration of 4000 cells/µL, (ii) each capsule was sealed within a diffusion chamber and cultured overnight in basal media + 100 ng/mL BMP2, (iii) one cell-laden capsule was implanted per mouse along with an acellular control capsule in a separate diffusion chamber, (iv) micro-computed topography (µCT) was performed 8-weeks post subcutaneous implantation to assess bone formation within the diffusion chambers, and (v) explanted capsules were processed and sectioned for histological assessment. (b) Mouse prior to subcutaneous implantation of diffusion chambers. Scale bar = 2 cm. (c) Mouse following subcutaneous implantation of diffusion chamber containing either cell-laden (TF+) or acellular (AC) capsules. Scale bar = 2 cm. (d) 3D view of whole explanted TF+ cell-laden (green) and acellular (purple) capsules by µCT 8-weeks post subcutaneous implantation. Tissue density thresholds were applied, identifying mineralisation densities >0.05 g/cm^3^ (blue) and >0.25 g/cm^3^ (yellow). Figures were generated using Imalytics Preclinical software v2. Scale bars = 500 µm. (e) Quantification of mineral density (g/cm^3^) recorded in TF+ cell-laden (green) and acellular (purple) scaffolds. Data shown as minimum, maximum, and mean density recorded in each of the eight capsules (four cell-laden and four acellular). (f) Alizarin Red staining of explanted TF+ cell laden and acellular alginate/chitosan polysaccharide capsules 8-weeks post subcutaneous implantation in vivo. One cell-laden and one acellular scaffold was implanted per mouse. Whole capsule scale bars = 500 µm, ×20 scale bars = 100 µm.

To investigate the in vivo functionality of SNA-enriched populations, we performed a preliminary investigation to evaluate bone formation in TF+ cell-laden scaffolds within diffusion chambers, after 8 weeks post-implantation in a subcutaneous implant mouse model.^62^ Up to 200,000 freshly isolated TF+ cells were obtained from each of four patient samples and encapsulated within alginate/chitosan polysaccharide capsules (4000 cells/µL), as per previous reports.^63,64^ Acellular capsules were prepared as a negative control to assess mineralisation of the scaffolds in vivo. Capsules were cultured overnight in basal media + 100 ng/mL BMP2 and sealed within diffusion chambers before subcutaneous implantation in four male Balb/c athymic mice (Figure S13).^65^ After 8 weeks, mice were sacrificed, and micro-computed topography (µCT) was performed on the explanted scaffolds (Figure 5(a)–(c)). While there was limited high density bone formation (~0.75 g/cm^3^) across the cell-laden scaffolds, regions of low-density mineralisation (density >0.05 g/cm^3^) were detected in all TF+ cell-laden capsules (Figure 5(d)). Tissue with a density >0.25 g/cm^3^ shown in yellow. The highest density mineralisation was measured in the TF+ cell-laden scaffold implanted in mouse 3 (0.72 g/cm^3^) (Figure 5(e)). In contrast, all acellular scaffolds displayed a density <0.05 g/cm^3^, confirming the alginate/chitosan polysaccharide capsules did not mineralise in vivo after 8 weeks. Furthermore, Alizarin Red staining of bone nodule formation confirmed increased calcification in TF+ cell-laden capsules, while no positive staining was observed in acellular controls (Figure 5(f)).

## Discussion

In this study, we examined the transcriptomic signatures, and the in vitro and in vivo functionality of enriched SSC populations, assessed in over 100 patient samples. We utilised scRNA-seq for the profiling of human bone marrow sub-populations, and employed SNAs to select cells expressing candidate mRNA targets. We introduce a new methodology for SSC enrichment from human bone marrow and describe novel markers that characterise functional populations of skeletal progenitors.

Initial profiling of a large scRNA-seq human bone marrow dataset described expression of *CXCL12*^48^ and *LEPR*^4^ within the pericyte cluster, well classified markers of the bone marrow stromal niche. Further analysis detected differential expression of *SPARCL1* within the stromal compartment. Given that SPARCL1 has been: (i) previously shown to activate BMP/TGF-β signalling to promote the differentiation of C2C12 cells in mice,^66^ (ii) is highly homogenous to bone regulatory protein SPARC/OCN,^67^ and (iii) SPARCL1 protein has been evidenced as a regulator of the ERK/MEK signalling pathway^68^; new SNAs were designed against *SPARCL1* mRNA to investigate *SPARCL1* as a new target for SSC enrichment.

In addition to *SPARCL1*, we designed SNAs to target a wide panel of candidate SSC markers identified within the literature. Our research employed the CFU-F assay for in vitro functional validation of enriched populations; considered a gold-standard assay for determining the clonogenic capacity of bone marrow derived SSC populations since the seminal studies of Friedenstein et al. and colleagues.^45,69–71^ In addition to high CFU-F enrichment observed in SPARCL1+ populations, CD200, CD146 and SOST were identified as candidate targets for SSC enrichment. These results are consistent with previous findings which identify CD200+^8,72^,*CD146*+^2,36,73^ as viable targets for SSC isolation. However, limitations to both populations have been identified; CD200 mRNA displayed poor SSC-specificity,^74,75^ while SSCs have been identified in both CD146+ and CD146−/low fractions.^76,77^ Therefore, these markers are not suitable for the use as lone mRNA targets but are a valuable resource to be used in conjunction with other markers to purify a pool of SSCs. Elevated CFU-F enrichment in SOST+ populations is interesting, given Sclerostin is widely accepted as an osteocyte-specific protein.^78^ However, *SOST* RNA has been described in several other cell types as a key regulator of Wnt signalling and consequently functions in a number of key pathways; including, stem cell proliferation, osteoblast and chondrocyte activity and mechanical response.^79–82^

Following the initial identification of SNA targets for SSC enrichment, we sought to deliver purified populations into the scRNA-seq pipeline. The scarcity of SSC within human bone marrow, <1 in 100,000 of BM-MNCs,^61^ presents a significant challenge for transcriptomic profiling of SSCs. Limitations of scRNA-seq protocols in detecting cell types with a frequency less than 1% have been recently reported as these cells typically appear as outliers.^83^ Therefore, pre-sorting cells prior to downstream scRNA-seq can increase read depth by reducing competition for sequencing capacity between cells of interest and other cell types.^84^ Sequencing of populations enriched for desirable cell types, has been applied previously for the characterisation of rare cell haematopoietic subtypes and identification of unique biomarkers.^85,86^ Thus, the Drop-seq data using CD146, CD200, *SOST* and *SPARCL1* SNAs, together with a Stro-1+ population, produced transcripts from 1521 cells from an initial 200 million bone marrow cells. ScRNA-seq revealed a second-generation panel of candidate SSC markers; including *TF, DCN* and *CALD1*, which served as SNA targets and demonstrated enhanced CFU-F enrichment when used both alone and in conjunction to *SPARCL1*-targetted SNAs. We further confirmed elevated expression of the candidate SSC markers in stromal/skeletal progenitor populations, through analysis of publicly available scRNA-seq data, which included scRNA-seq of human and mouse bone marrow. Additionally, we found no significant differential expression of our markers across osteogenic and adipogenic precursor populations (in LEPR+CD45− SSCs),^52^ suggesting the candidate markers are expressed by SSCs independent of early lineage commitment. A role of *DCN* in skeletal development has been identified previously; high expression has been reported in a variety of cells, including osteoblasts, perivascular chondrocytes and throughout the bone periosteum.^87^ Additionally, DCN can function in cascades, regulating cell differentiation, angiogenesis, and bone mineralisation.^88–93^ Similarly, the essential role of TF in iron-delivery and consequently, cell growth and survival, correlates with highly proliferative cell types and anti-apoptotic activity.^94–97^ Finally, CALD1 functions in calcium-mediated signalling pathways in bone,^98^ and its expression in BMMNCs has been reported previously.^99^

These targets were shown to be unsuitable candidates for classical MACS cell sorting based on surface epitope expression. These findings suggest targets identified by scRNA-seq better complement mRNA-based SNA strategies, reflective of the lack of correlation between mRNA levels and protein abundance.^100–102^ This limitation was previously described by Fitter et al. who identified no correlation between *HSPA8* (mRNA) and Stro-1 (surface protein).^42^

Across all candidate markers, the most consistent CFU-F enrichment was observed in TF+ populations and achieved the highest CFU-F counts when used in conjunction with *SPARCL1* SNAs. Integral to classification of the TF+ population as an enriched SSC population is in vivo functionality. In a preliminary in vivo investigation, we demonstrated how TF+ cell-laden scaffolds produced mineralisation, absent in acellular scaffolds. We applied stringent conditions to assess bone formation in TF+ enriched SSC populations; scaffolds were enclosed with diffusion chambers to prevent confounding results caused by potential infiltration of endogenous cell populations, and the subcutaneous implant mouse model provides an environment absent of osteogenic cues; testament to the formation of mineralised tissue in TF+ enriched cell-laden scaffolds recorded. However, this study presents an elementary approach to assess the regenerative capacity of TF+ cells. Future studies would look to recapitulate the fracture environment and assay the full in vivo tri-lineage capacity, while serial transplantation studies will facilitate full assessment self-renewal capacity of the *TF+* population.^103^

Future investigations also warrant the acknowledgement of limitations in the current study design. While we evidence enhanced CFU-F enrichment in SNA-selected populations, we do not observe purified populations and a high degree of heterogeneity persisted within the scRNA-seq data. This is likely due to the use of a single marker for SSC enrichment. In the current study, we describe cell-sorting approaches using Cy5 and JOE SNAs in parallel to elevate CFU-F capacity. Future studies seek to determine the combination of SNA targets that offer the highest level of SSC purity and validate these findings in vitro and in vivo. However, it is fundamental to note that with increased specificity of SSC enrichment, comes reduced total cell number obtained. Pseudotime inference analysis can be employed to map the developmental trajectory of cells, which in the case of SSCs can support the characterisation of subtypes within enriched or expanded SSC populations.^104,105^ Unfortunately, we were unable to perform these workflows in the current study due to the low frequency of bone marrow SSCs, warranting scRNA-seq of bone marrow from an increased numbers of donors in future studies. Limited cell numbers also restrict current methods of in vitro analysis as we were unable to obtain sufficient cell numbers to perform quantitative analysis of tri-lineage differentiation in vitro (RT-qPCR). Furthermore, assessment of the enriched SSC population should evaluate marker expression and SSC isolation in the full context; flow cytometry to detect expression of surface markers used to characterise the growth plate SSCs would support comparison of SSCs from distinct sources.^1,9^

In summary, the current study harnesses, for the first time, Drop-seq as a powerful tool for the parallel single-cell sequencing of human adult bone marrow for SSC enrichment. The novel markers identified serve as targets for innovative SSC enrichment using SNAs to identify target mRNA. The enriched populations display the capacity for colony formation and tri-lineage differentiation in vitro, and using *TF*+ as an elementary population, we evidence formation of mineralised tissue in vivo. The findings describe new avenues for development of SSC isolation protocols, a valuable resource for future development of SSC-based therapies in the treatment of skeletal damage and disease.

## Materials and methods

### Subjects and samples

Bone marrow samples were obtained from haematologically healthy patients undergoing hip replacement surgery under local ethics committee approval (ERGO 31875, REC Ref. 18/NW/0231, IRAS project ID 234701).

### Human bone marrow tissue processing

Human bone marrow was washed in α-MEM medium and passed through a 70 µm cell strainer. Only samples intended for incubation with SNAs were treated with RosetteSep Human Granulocyte Depletion Cocktail (StemCell Technologies, Cambridge, UK) adding 100 µL to bone marrow resuspended in 5 mL α-MEM medium for 20 min. All samples were subjected to density centrifugation using Lymphoprep™ (StemCell Technologies, Cambridge, UK). The buffy coat layer, containing BM-MNCs was washed in basal medium (α-MEM containing 10% FBS and 100 U mL−1 penicillin and 100 µg/mL^−1^ streptomycin; Lonza). Cells were subsequently either sorted using magnetic activated cell sorting (MACS) or incubated with SNAs for FACS sorting.

### Enrichment of CD45−CD146+ skeletal progenitor, CD144+ endothelial and CD144−/CD106+ pericyte cell populations

Whole bone marrow was diluted 1:1 with α-MEM (Gibco) digested with collagenase IV (20 U/mL, Thermo Fisher, 17104019) for 30 min at 37°C under continuous rotation. Subsequently, BM-MNCs were isolated as described previously.^60^ Where indicated, MACS was conducted to enrich or deplete cells. Up to a total of 1 × 108 BM-MNCs were used for each separation. Cells expressing CD45 (Miltenyi Biotec, 130-045-801), CD146 (Miltenyi Biotec, 130-093-596), CD144 (Miltenyi Biotec, 130-097-857), CD106 (Miltenyi Biotec, 130-104-123 and 130-048-102) were isolated using Large Separation (LS) columns (Miltenyi Biotec, 130-042-401) according to manufacturer’s instructions. Each of the three patient samples were sorted into three distinct populations: CD45−/CD146+, CD144+ and CD144−/CD106+ cells.

### Enrichment of Stro-1+ skeletal progenitors

Bone marrow cells were initially incubated with blocking buffer (α-MEM, 10% human serum, 5% FCS and 1% bovine serum albumin) and subsequently with primary Stro-1 antibody (undiluted hybridoma culture supernatant.^60^ Following washes in buffer (2 mM ethylenediaminetetraacetic acid (EDTA) and 1% BSA in phosphate buffered saline); cells were incubated with magnetic bead-conjugated secondary antibody (Miltenyi Biotec). After further washes, Stro-1 positive cells were collected by MACS according to manufacturer’s instructions (Miltenyi Biotec).

### Enrichment of Decorin+ and Transferrin+ cells (Surface Antigen Detection)

Surface antigen detection of Decorin and Transferrin was performed using MACS. Ten million granulocyte-depleted marrow cells were initially incubated with blocking buffer (α-MEM, 10% human serum, 5% FCS and 1% bovine serum albumin) for 15 min at 4°C, then with primary antibody against Decorin (Abcam, ab181456) or Transferrin (Bio-techne, NBP2-02264) at 1/30 dilution for 30 min at 4°C. Following washes in buffer (2 mM ethylenediaminetetraacetic acid (EDTA) and 1% BSA in phosphate buffered saline), cells were incubated with magnetic bead-conjugated secondary antibody (Miltenyi Biotec) for 15 min at 4°C. After further washes, MACS positive and negative cells were collected by MACS according to manufacturer’s instructions (Miltenyi Biotec). For CFU-F assessment, cells were plated in a 12-well plate at 50,000 cells per well for MACS positive and negative cells and 5000 cells per well for unsorted cells.

### Synthesis of gold nanoparticles (AuNPs)

For the synthesis of spherical gold nanoparticles, a modified Turkevich approach was followed. Briefly, a solution of sodium tetracholoroaurate (100 mL, 1 mM) was brought to the boil whilst stirring (700 rpm). To this, a sodium citrate solution (5 mL, 2% wt) was added and a colour change from yellow to colourless to purple was observed until a final solution colour of wine red was established, indicating the successful formation of nanoparticles. The solution was left to stir for an additional 15 min and subsequently cooled to room temperature under slow stirring (200 rpm). Once cooled, citrate ligands on the surface of the nanoparticles were exchanged with bis-sulfonatophenyl phosphine dehydrate dipotassium salt (BSPP) by adding 20 mg to the solution. After stirring overnight, a concentrated solution of NaCl (~1.5 mL) was added until a colour change to purple/blue was observed indicating particle aggregation via charge screening. Particles were then purified by two sets of centrifugation (5000 rpm, 15 min) and re-dispersed in Milli-Q water. Remaining large aggregates were subsequently purified by filtration (0.2 µm, VWR) and particles were stored at 4°C prior to further functionalisation.

### Synthesis of spherical nucleic acids (SNAs)

BSPP coated spherical AuNPs (1 mL, 10 nM) in Milli-Q water were incubated with thiol modified oligonucleotides (1 mL, 3 µM) overnight to allow the mixture to equilibrate. (For oligonucleotide synthesis see supplemental text). BSPP (10 µL, 1 mg/20 µL), phosphate buffer (0.1 M, pH 7.4) and sodium dodecyl sulphate (SDS) (10%) were added to the spherical AuNP/oligonucleotide solution to achieve a final concentration of 0.01 M phosphate and 1% SDS respectively. The NaCl concentration was brought up to 0.3 M over an 8 h period in a stepwise manner by the gradual addition of NaCl. The solution was sonicated (5 min) after every addition to keep the particles well-dispersed during the salting procedure. Following the NaCl additions, the final solution was shaken for an additional 16–20 h to yield fully functionalised AuNPs. To remove any unbound oligonucleotides, the sample was purified by three rounds of centrifugation (16,400 rpm, 20 min) including supernatant removal and resuspension in phosphate buffer saline (PBS).

For flare hybridisation, previously synthesised SNAs (16 nM, 500 µL) were incubated with an excess of partially complimentary flare oligonucleotides (960 nM, 500 μL). The solution was then heated to 70°C for 5 min followed by slow cooling to room temperature. Excess flare strands were purified from the solution by two rounds of centrifugation (16,400 rpm, 15 min) and redispersed in PBS. The solution was then stored at 4°C prior to further use.

### FACS

SNA treated cells were collected from human bone marrow using a FACS Aria cytometer (Becton Dickinson, Wokingham, UK). In total, 20 million cells were sorted from each patient sample. Cells were gated for monocytes, single cells, and Cy5 fluorescence, with the top 20% of fluorescent cells collected. Positive samples were determined to be within the top half of the Cy5 fluorescent cells and negative samples were considered to be within the bottom half of the collected cells. For studies using Cy5 and JOE SNAs in combination, a four-way gating system sorted Cy5+/JOE+, Cy5−/JOE+, Cy5+/JOE−, Cy5−/JOE− cells, with only the top 10% of Cy5+/JOE+ collected for plating. Data were analysed on Flowing Software version 2.5 (http://www.flowingsoftware.com). Collected cells were subsequently used for CFU-F quantification, cellular differentiation assays, or Drop-seq.

### Colony forming units-fibroblast (CFU-F) assay

CFU-F assessment was conducted to demonstrate colony growth.^10,106^ Positive or negative fraction cells were seeded at limiting dilution into each well of 12- or 6-well tissue culture plates (density 102–103 cells /cm^2^). Cells were grown for 14 days, with a medium change after 7 days. On day 14, wells were washed with PBS and then fixed with 95% EtOH for 10 min. The wells were air-dried, and 1 mL of 0.05% crystal violet solution was added to each well for 1 min. The wells were washed twice with distilled water and the number of stained colonies determined.

### Drop-seq of human bone marrow cells

Drop-seq was performed as previously described^33^ and outlined in the online Drop-seq Laboratory Protocol version 3.1 (http://mccarrolllab.org/dropseq) with any adjustments described below. Drop-seq samples were processed as two separate experiments, with the first including the CD45−/CD146+, CD144+ and CD144−/CD106+ populations and the second including the SNA-Cy5+ and Stro1+ populations (which for the purpose of clarity, will be known as Drop-seq1 and Drop-seq2 respectively). In brief, droplet microfluidic devices, according to Macosko et al.,^33^ were fabricated in poly(dimethylsiloxane) (PDMS) and functionalised incubating channels with 1% trichloro(1H,1H,2H,2H-perfluoro-octyl)silane (Sigma-Aldrich, 448931) in HFE-7500 (3 M) for 5–10 min at RT. Cells were co-encapsulated with Drop-seq beads (ChemGenes: lot 083117) in 1 nL droplets using 15000 mL/hr oil (QX200, Biorad), 4000 mL/hr cell suspension and 4000 mL/hr bead flow rates (droplet generation frequency 2 kHz), using an open instrumentation microfluidic workstation (https://dropletkitchen.github.io/). Following cell lysis, the droplet emulsion was broken using perfluoro-1-octanol (Sigma-Aldrich) to generate single-cell transcriptomes attached to microparticles (STAMPs). cDNA synthesis was conducted using Maxima H Minus Reverse Transcriptase (Thermo Fisher Scientific). This was followed by PCR amplification (Kapa HiFi Hotstart ReadyMix) using 4+15 PCR cycles for Drop-seq1 and 4+14 cycles for Drop-seq2 (95°C 3 min − 4 cycles of: 98°C 20 s; 65°C 45 s; 72°C 3 min – 15/14 cycles of: 98°C 20 s; 67°C 20 s; 72°C 3 min – 72°C 5 min; 4°C hold). Libraries were tagmented (Nextera XT DNA Library Preparation Kit, Illumina) and amplified before pooling samples for paired-end sequencing using a NextSeq 500/550 High Output Kit v2.5 (Illumina, 20024906) and NextSeq 500 system (Illumina).

### Sequence alignment

Raw sequencing reads were aligned following the Drop-seq Core Computational Protocol (Drop-seq tools v1.0, http://mccarrolllab.org/dropseq/) using STAR.^107^ Sequencing data was aligned to the human hg19 reference genome (GSM1629193). Raw sequencing reads were demultiplexed, grouping reads by cell barcode to generate a digital gene expression (DGE) matrix for downstream analysis, using Drop-seq tools (v1.0). A modified multi-mapper pipeline was executed to correct multiple alignment.^39^

### Data clustering

Analysis of the Drop-seq1 and Drop-seq2 datasets was conducted using the software R (version 3.5.0) according to the standard pipeline of functions included in the R Seurat package (version 4.0) (http://satijalab.org/seurat/). Drop-seq1 and Drop-seq2 datasets were processed and analysed separately.

### Drop-seq1

The Drop-seq1 dataset, comprising 26,657 cells, was subjected to a number of quality control measures: filtering out low-quality cells (cells expressing <200 genes or >10% mitochondrial genes) and potential cell doublets (cells expressing >4500 genes). DoubletFinder (v3) identified and removed 155 potential doublets from subsequent analyses.^108^ Cells were subsequently normalised using the function; sctransform. Sample integration was performed according to the Seurat SCTransform integration workflow.^109^ Linear dimensionality reduction was performed using Principle Component Analysis (PCA) and non-linear dimension reduction using Uniform Manifold Approximation and Projection (UMAP) using 25 Principle Components (PCs). Clustering analysis was conducted using the Seurat function, FindClusters, at a resolution of 1 and default parameters, revealing 31 clusters. To assign identifies to the clusters, the function, FindAllMarkers, was performed, employing the non-parametric Wilcoxon rank sum test for characterisation of significant cluster-specific gene expression for comparison against previously described biomarkers for bone marrow cell populations. Differential gene expression analysis revealed eight distinct cell types. Pericyte cells were defined by expression of *LEPR, CXCL12, VCAM-1* and *ANGPT1*.

### Drop-seq2

For the Drop-seq2 dataset, comprising 1900 cells, quality control thresholds were further refined due to the reduced number of cells. Cells expressing >10% mitochondrial RNA, or <200/>2600 unique reads were filtered from the dataset. DoubletFinder^108^(v2.0) identified a further 22 potential doublets, none of which were present within the skeletal progenitor cluster, and were subsequently filtered from the dataset prior to downstream analysis. Following the removal of undesirable cells, the dataset contained 1573 cells. Sample integration was performed according to the Integration workflow Seurat SCTransform integration workflow.^109^ Linear dimensionality reduction was performed using PCA and non-linear dimension reduction using UMAP and 16 PCs. Clustering was conducted using the Seurat function, FindClusters, at a resolution of 1.5 and default parameters. This revealed 16 clusters, which were broadly classified into eight cell types by differential gene expression analysis using FindAllMarkers. The skeletal progenitor cluster was defined by expression of *CXCL12, LEPR*, CD200 and *SPARCL1*.

### Analysis of publicly available scRNA-seq data of human and murine BMMNCS

To evaluate the expression of target markers in bone marrow data, publicly available data was sourced. Data from Wang et al. and colleagues, profiling CD271^+^ BMMNCS was acquired from GEO database under the accession number GSE147287. Data was processed and analysed according to the authors description.^52^ We also explored an integrated dataset of mouse bone marrow niche scRNA-seq data, available through the Open Science Framework (https://osf.io/ne9vj).^53^

### Osteogenic differentiation assay

Passage 1 cells were cultured in basal media at 37°C in 5% CO_2_ until confluent, seeded at 10,000 cells per well on a 12-well plate and subsequently cultured in basal media for 24 h. Cells were then cultured in osteogenic media (basal medium with 50 µM ascorbic acid 2-phosphate, 10 nM Dexamethasone and 10 nM vitamin D3) for 14 days at 37°C in 5% CO_2_ with media change every 3–4 days. Cells were washed in PBS, fixed in 95% EtOH and stained with alkaline phosphatase.

### Adipogenic differentiation assay

Passage 1 cells were cultured in basal media at 37°C in 5% CO_2_ until confluent, seeded at 10,000 cells per well on a 12-well plate and subsequently cultured in basal media until 80% confluent, after approximately 3–5 days. Cells were cultured in adipogenic media (basal medium with 100 nM dexamethasone, 500 µM IBMX, 3 µg/mL ITS solution, and 1 µM rosiglitazone) for 14 days at 37°C in 5% CO_2_ with media change every 3–4 days. Cells were washed in PBS, fixed in 4% paraformaldehyde, washed in PBS again and stained with Oil Red O.

### Chondrogenic differentiation assay

Passage 2 cells were cultured in basal media at 37°C in 5% CO_2_ until confluent, then diluted to 500,000 cells per mL in chondrogenic media (α-MEM containing 100 U mL−1 penicillin and 100 µg/mL−1 streptomycin, 100 µM ascorbic acid 2-phosphate, 10 ng/mL TGF-B3, 10 µg/mL Insulin-Transferrin-Selenium (ITS) solution, 10 nM dexamethasone) in a universal container. Cells were centrifuged at 400× *g* for 10 min to form a cell pellet, and all but 1 mL of media removed. Cells were cultured with the tube cap loose for 14 days at 37°C in 5% CO_2_ under hypoxic conditions. The media was changed every 2 days. Cells were washed in PBS, fixed in 95% EtOH. Fixed pellets were dehydrated through graded 45 min treatments of EtOH (50%–100%) and Histoclear (100%). Samples were embedded in molten paraffin wax (Fisher Scientific, UK, 12624077) and sections cut at 7 µm on a Microm330 microtome (Optec, UK) and mounted onto pre-heated glass slides. Slides were subsequently stained with Alcian blue and Sirius red.

### Preparation of alginate/chitosan polysaccharide capsules for cell-encapsulation and in vivo subcutaneous implantation

Alginate/chitosan polysaccharide capsules were prepared as previously described.^63,64^ Briefly, alginate solution was freshly prepared by dissolving 0.2 g Ultrapure alginate (NovaMatrix, Drammen, Norway), 0.3 g di-sodium hydrogen orthophosphate (210 mM) and 0.9 g sodium chloride in 10 mL dH2O and mixed thoroughly for 2 h prior to cell encapsulation. Chitosan (3 g) (Sigma-Aldrich, 448877) was added to 200 mL dH2O, 3 mL acetic acid and 1 g calcium chloride (50 mM). TF+ cells, collected by FACS following SNA incubation, were pelleted and the appropriate amount of alginate was added for a desired concentration of 4000 cells/µL alginate. Cell-laden or acellular alginate solution was vortexed for through mixing. The alginate was added dropwise to a petri dish containing chitosan solution and left for 45 min for formation of chitosan shell. Capsules were formed as either 25 µL or 50 µL droplets, containing 100,000 or 200,000 cells respectively. Capsules were washed three times in plain αMEM and cultured overnight in basal media supplemented with 100 ng/mL BMP2 before implantation. Alginate/chitosan polysaccharide capsules, with and without TF+ enriched populations, were sealed within diffusion chambers (Merck, UK, HAWP01200 and PR0001400) for subcutaneous implantation in mice, as described previously.^60^

### In vivo assessment of bone formation

All animal procedures were performed and approved under licence (P961B16FBD) in accordance with the regulations as laid down in the Animals (Scientific Procedures) Act 1986 and in accordance with institutional guidelines. Scaffolds were implanted subcutaneously in male athymic immuno-deficient HsdOla:MF1-Foxn1nu mice (29–38 g, 13–15 weeks old, Envigo, UK). Mice were housed in separate ventilated cages with access to food and water ad libitum. Diffusion chambers, containing alginate/chitosan polysaccharide capsules, with and without TF+ enriched populations, were implanted subcutaneously in mice. Up to two cell-laden and two acellular diffusion chambers were implanted per mouse. Chambers were implanted using sterile forceps into pockets created by blunt dissection within the sub-cutis layer on both flanks, as outlined previously.^60^ Incisions were closed with sterile resorbable sutures (Z148H, Ethicon, Johnson & Johnson Medical). Animals were monitored continuously for 8 weeks. After 8-weeks, the mice were euthanised by rising CO_2_ (2 l/min) and cervical dislocation. Diffusion chambers were harvested, and the polysaccharide capsules were explanted for micro-CT scanning and fixation for histological analysis.

### Micro-CT analysis

Micro-CT was performed using a MILabs OI-CTUHXR preclinical imaging scanner (Utrecht, The Netherlands). Mice were scanned at 4- and 8-week post-implantation of the scaffolds. At 4-week scans, mice were anesthetized by induction at 4% isoflurance (VetTech) and maintained at 1%–3% isoflurane and oxygen rate ~1.5 l/min. Four-week scans were conducted at 55 kV, 0.17 mA, 75 ms exposure, 0.25 rotation step, and an aluminium filtre (AI) of 500 µm. The scanning bed was set to 34°C–36°C and Lubrithal (Dechra) was applied before imaging to ensure no drying of the eyes throughout the scan. At 8-weeks post-implantation, mice were scanned post-euthanasia (50 kV, 0.21 mA, 75 ms exposure, 0.25 rotation step, 500 µm AI). To obtain higher quality images, samples were explanted and scanned (50 kV, 0.21 mA, 65 ms exposure, 0.25 rotation step, and an aluminium filtre (AI) of 100 µm). Micro-CT reconstructions were obtained *via* MILabs software (MILabs-Recon v. 11.00). 4- and 8- weeks micro-CT images were reconstructed at a voxel size of 40 µm^3^ and ex vivo scaffolds were reconstructed at 20 µm^3^. Formation of skeletal tissue was assessed using Imalytics Preclinical software v2.1 (Gremse et al. -IT GmbH).^110^ Two bone density phantoms (0.25 and 0.75 g/cm^3^ bone densities) were scanned at each time point using the same parameters to be used as a reference for quantification of bone density.

### Histological analysis of explanted in vivo samples

For histological analysis, in vivo samples were explanted and fixed in 50% alcohol formaldehyde (1% CaCl^2^) overnight at 4°C. Samples were dehydrated through graded EtOH (50%–100%) and Chloroform (100%) for 1 h at each stage. Sections were cut at 7 µm on a Microm330 microtome (Optec, UK) and mounted onto pre-heated glass slides. To visualise formation of mineralised nodules, sections were stained with Alizarin Red S solution (pH 4.2; Sigma-Aldrich, A5533 in 5% CO_2_ in 24-well plates and imaged using a Zeiss Axiovert 200 inverted microscope with an Axiocam) for 2 min.

### Microscopy

Cells were cultured at 37°C in 5% CO2 in 24-well plates and imaged using a Zeiss Axiovert 200 inverted microscope with an Axiocam MR camera for fluorescent imaging and Axiovert HR camera for brightfield imaging operated by Zeiss Axiovision software version 4.7.

### Statistical analyses

Wilcoxon-Mann-Whitney statistical analysis and ANOVA were performed where appropriate using the SPSS for Windows program version 23 (IBM Corp, Portsmouth, Hampshire, UK). Data presented as mean ±95% confidence limits and significance was determined with a p-level of 0.05 or lower.

## Supplemental Material

sj-docx-1-tej-10.1177_20417314231169375 – Supplemental material for Single-cell RNA-sequence analysis of human bone marrow reveals new targets for isolation of skeletal stem cells using spherical nucleic acids Click here for additional data file. Supplemental material, sj-docx-1-tej-10.1177_20417314231169375 for Single-cell RNA-sequence analysis of human bone marrow reveals new targets for isolation of skeletal stem cells using spherical nucleic acids by Elloise Z Matthews, Stuart Lanham, Kate White, Maria-Eleni Kyriazi, Konstantina Alexaki, Afaf H El-Sagheer, Tom Brown, Antonios G Kanaras, Jonathan J West, Ben D MacArthur, Patrick S Stumpf and Richard OC Oreffo in Journal of Tissue Engineering
