# Supplementary material for: Single-cell RNA-sequence analysis of human bone marrow reveals new targets for isolation of skeletal stem cells using spherical nucleic acids
Source: J Tissue Eng. 2023 May 16;14:20417314231169375. doi: 10.1177/20417314231169375 (PMC10192814; doi:10.1177/20417314231169375)
Supplement: sj-docx-1-tej-10.1177_20417314231169375 – Supplemental material for Single-cell RNA-sequence analysis of human bone marrow reveals new targets for isolation of skeletal stem cells using spherical nucleic acids [file sj-docx-1-tej-10.1177_20417314231169375.docx]

Supplementary Materials

**Single-cell RNA sequence analysis of human bone marrow reveals new targets for isolation of skeletal stem cells using spherical nucleic acids**

Elloise Z. Matthews, Stuart Lanham, Kate White, Maria-Eleni Kyriazi, Konstantina Alexaki, Afaf H. El-Sagheer, Tom Brown, Antonios G. Kanaras, Jonathan West, Ben D. MacArthur, Patrick S. Stumpf*^,^ and Richard O.C. Oreffo*.

*Corresponding authors Patrick S. Stumpf patrick_stumpf@web.de or Richard O.C. Oreffo [roco@soton.ac.uk](mailto:roco@soton.ac.uk)

Supplementary Text

Oligonucleotide synthesis

Standard DNA phosphoramidites, solid supports and reagents were purchased from Link Technologies and Sigma-Aldich. Solid support 3'-Thiol-Modifier C3 S-S CPG, 1000/110, item number: 2361, 5'-Fluorescein-CE Phosphoramidite (6-FAM), item number: 2134, Cy-5-CE phosphoramidite (Cyanine 650) item number: 2521 and 5’-Amine monomer (5'-TFA-Amino-Modifier C6-CE Phosphoramidite item number: LK2124-F100 were purchased from Link Technologies Ltd. Automated solid phase synthesis of oligonucleotides was performed on an Applied Biosystems 394 synthesiser. Synthesis was performed on a 1.0 µmol scale involving cycles of acid-catalysed detritylation, coupling, capping, and iodine oxidation. Standard DNA phosphoramidites were coupled for 60 s and chemically modified phosphoramidites for 10 min. Coupling efficiencies and overall synthesis yields were determined by the inbuilt automated trityl cation conductivity monitoring facility.

5’-Amino modified oligonucleotides on resin was treated with a solution of diethylamine (10% in acetonitrile) for 20 min in order to selectively remove the cyanoethyl protecting groups.

The ONs were then cleaved from the solid support and protecting groups from the nucleobase and backbone were removed by exposure to conc. aqueous NH_3_ for 60 min at room temperature followed by heating in a sealed tube at 55 °C for 5 h for unmodified oligonucleotides and for 17 h at room temperature for the 5’-Cy5 modified oligonucleotides.

Oligonucleotides purification

The fully deprotected oligonucleotides were purified by RP-HPLC on a Gilson system using a Luna 10 μm C8(2) 100 Å pore Phenomenex column (250 × 10 mm) with a gradient of MeCN in aqueous triethylammonium bicarbonate (TEAB) (buffer A, 0.1 m TEAB, pH 7.5; buffer B, 50% buffer A in MeCN). Buffer B was increased 25–80% over 20 min at a flow rate of 4 mL⋅min^-1^ and elution was monitored by UV absorption between 260–295 nm.

N-Hydroxysuccinimide (NHS) labelling

### HPLC purified and freeze-dried 5-amino oligonucleotide (200 nmole) was dissolved in CO_3_^-/^HCO_3_^-^ buffer (0.5 M, pH 8.75, 120 μL) and mixed with the 6-JOE, SE NHS ester dye (from AAT Bioquest, Cat no.: 203), (50 eq) dissolved in DMSO (80 μL). The reaction mixture was shaken (750 rpm) at 25 °C for 4 h. The final solution was desalted and excess NHS ester reagent removed using a NAP^TM^-25 column (G.E. Healthcare, cat. no. GE17-0854-02) prior to RP-HPLC purification.

Mass Spectrometry of oligonucleotides

Mass spectrometry was recorded on a UPLC-MS Waters XEVO G2-QTOF (ESI^-^) using an ACQUITY UPLC ON BEH C18 column, 130 Å (1.7 µm, 2.1 mm × 50 mm). Data was then de-convoluted using MassLynx v4.1. A gradient of CH_3_OH in Et_3_N and hexafluoroisopropanol (HFIP) was used (buffer A, 8.60 mm Et3N, 200 mm HFIP in 5% CH_3_OH/H_2_O (v/v); buffer B, 20% buffer A in CH_3_OH). Buffer B was increased from 0–70% over 8 min, at a flow rate of 0.2 mL⋅min−1.


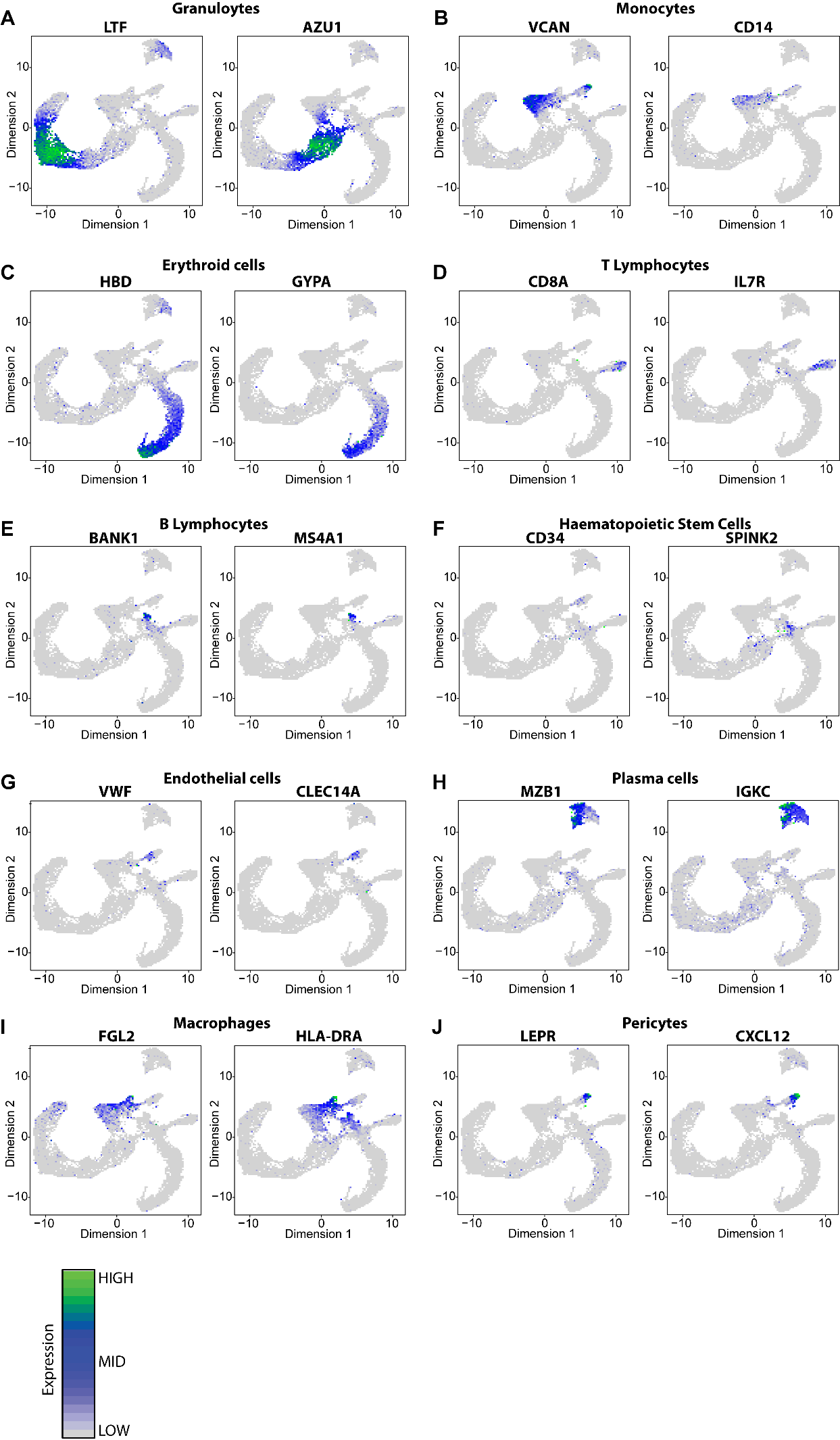


Fig. S1.

Use of lineage biomarkers to characterise Drop-seq data clusters from CD45-/CD146+ skeletal progenitor population, CD144+ endothelial cells, CD144-/CD106+ pericytes and unselected bone marrow cells into 10 cell subpopulations. **A)** Granulocytes, **B)** Monocytes, **C)** Erythroid cells, **D)** T Lymphocytes, **E)** B Lymphocytes, **F)** Haematopoietic stem cells, **G)** Endothelial cells**, H)** Plasma cells, **I)** Macrophages, **J)** Pericytes**.**


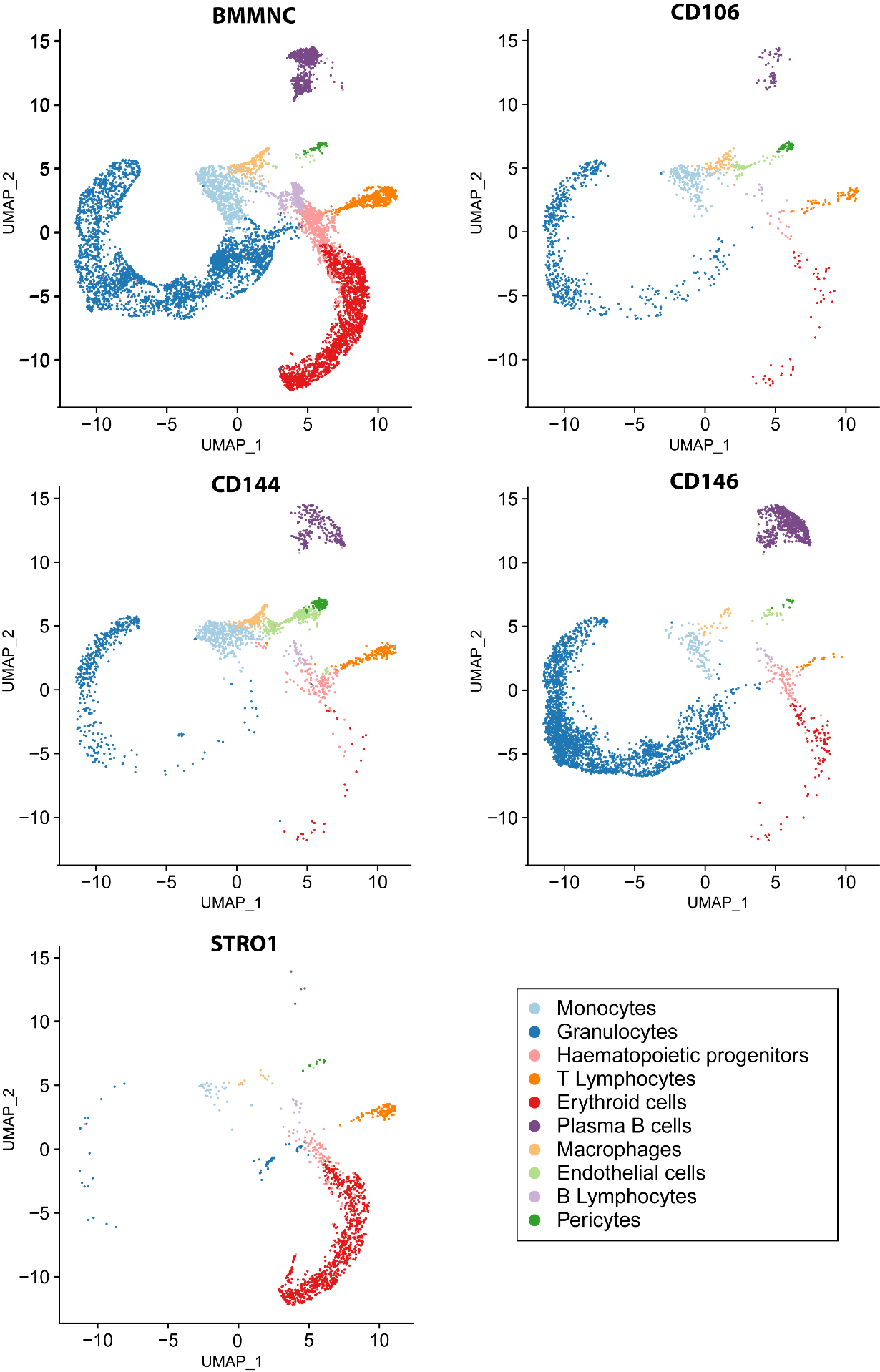


Fig. S2. Distribution of sequenced populations (CD144+, CD146+, CD106+, STRO1 and Unsorted bone marrow mononuclear cells (BMMNC)) across cell-type clusters on a UMAP plot.


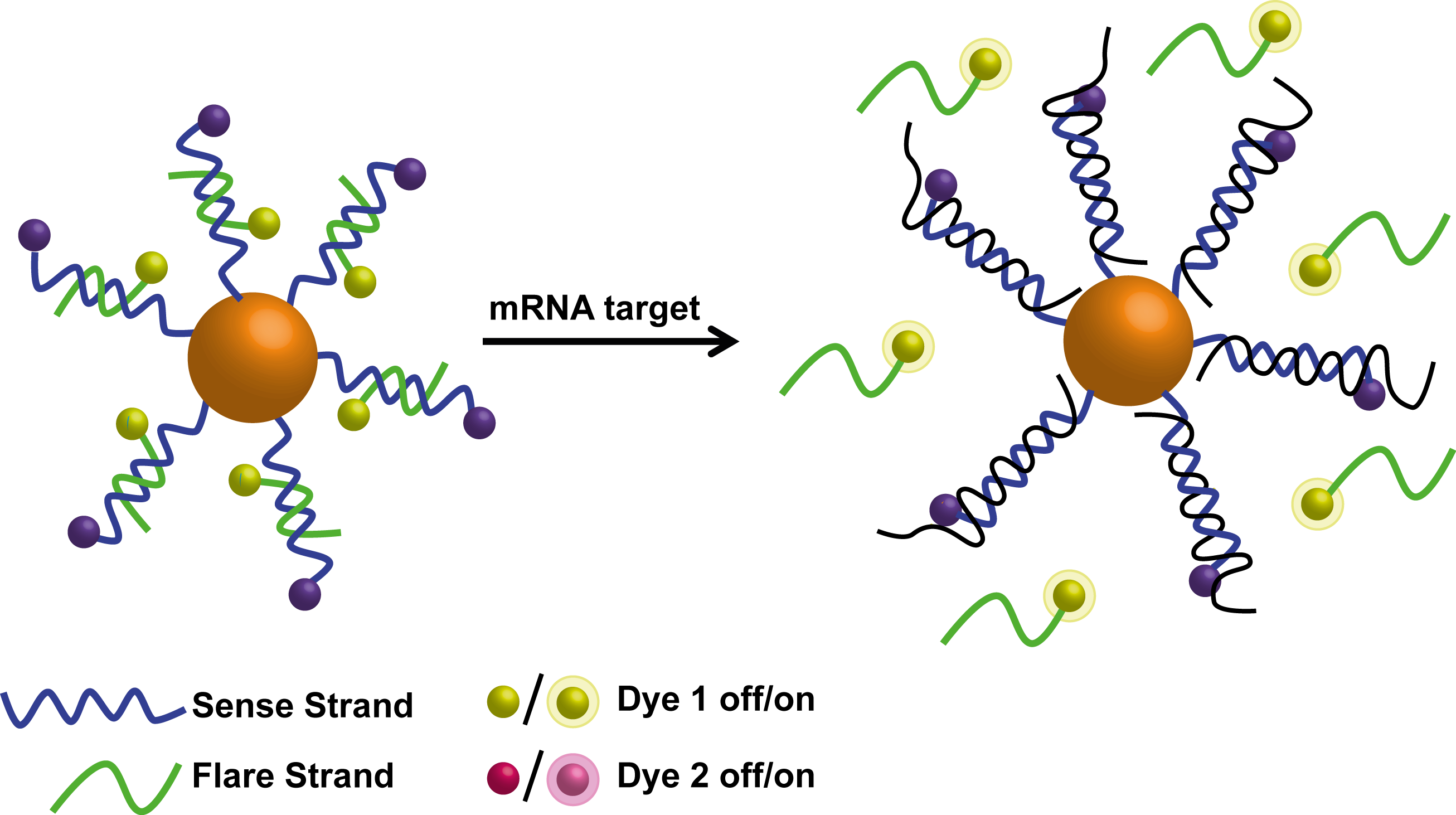


Fig. S3.

Schematic illustration of mRNA detection using SNAs. Upon hybridization of the flare to the sense strand the dyes on the oligonucleotide strands are quenched and a fluorescence signal cannot be detected due to their close proximity to the gold nanoparticle core. Upon detection of the specific mRNA target, the flare strand is displaced due to competitive hybridization and is no longer found in close proximity to the AuNP. The fluorescence of the flare strand is thus restored, resulting in a detectable fluorescence signal. The dye on the sense strand should remain quenched at all times and acts as control to ensure that the detectable fluorescence from the flare strand is due to mRNA recognition rather than SNA degradation by endocellular nucleases thus avoiding false positive results.


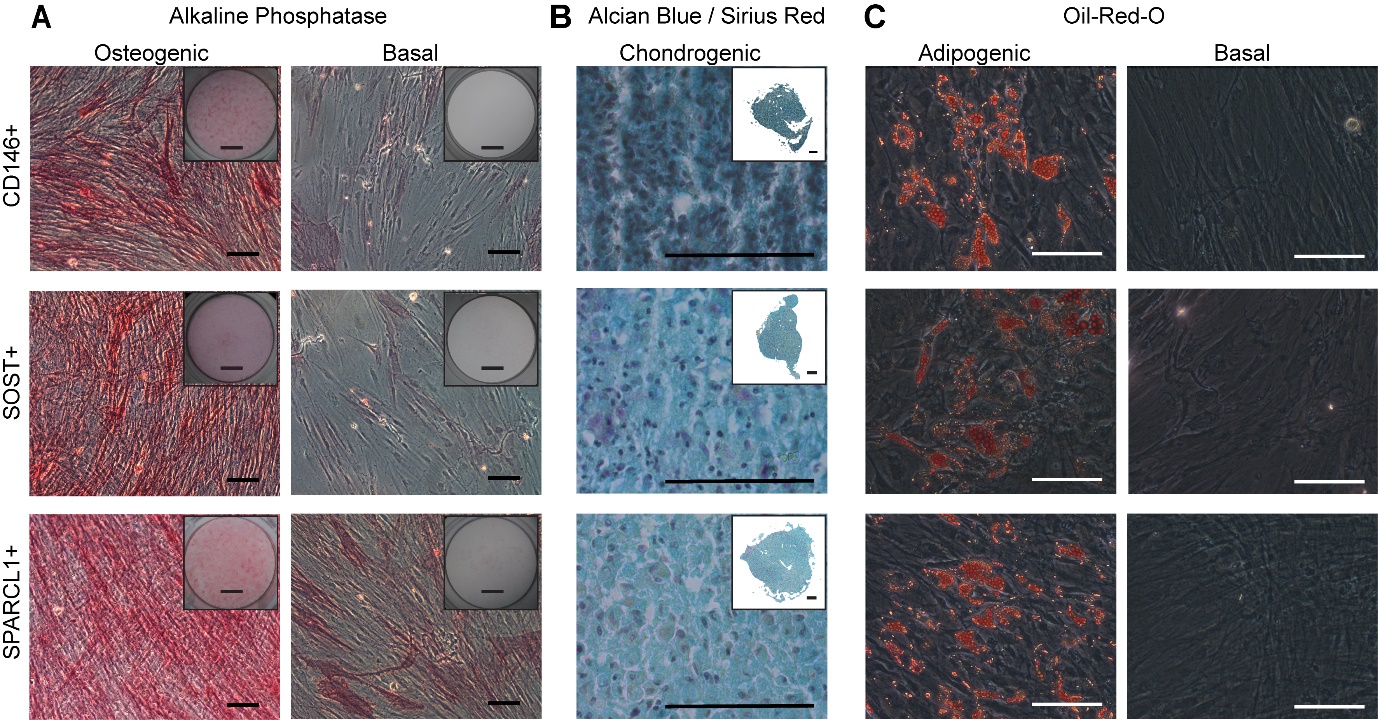


Fig. S4.

Trilineage differentiation of *CD146*+, *SOST+* and *SPARCL1+* enriched cell populations. Cells were collected using SNAs positively targeting CD146, SOST and SPARCL1 mRNA.

**A)** Osteogenesis promotion: Cells were cultured in basal medium with 50 µM ascorbic acid 2-phosphate, 10 nM dexamethasone and 10 nM vitamin D3 for 14 days. Osteogenesis was visualised with alkaline phosphatase staining. Scale bars = 100 µm (Whole well scale bar = 10 mm). **B)** Chondrogenesis promotion: Cells were cultured in basal medium supplemented with 100 µM ascorbic acid 2-phosphate, 10 ng/mL TGF-B3, 10 µg/mL ITS solution and 10 nM dexamethasone for 14 days. Alcian blue/Sirius Red staining revealed proteoglycan synthesis (Blue denotes proteoglycan deposition, red/purple indicates collagen deposition). Scale bars = 100 µm. **C)** Adipogenesis promotion. Cells were cultured in basal medium with 100 nM dexamethasone, 500 µM IBMX, 3 µg/mL ITS solution, and 1 µM rosiglitazone for 14 days. Oil-Red-O staining indicates lipid droplet formation. Scale bars = 100 µm. Results were obtained from 3 different patient samples; representative images are shown.


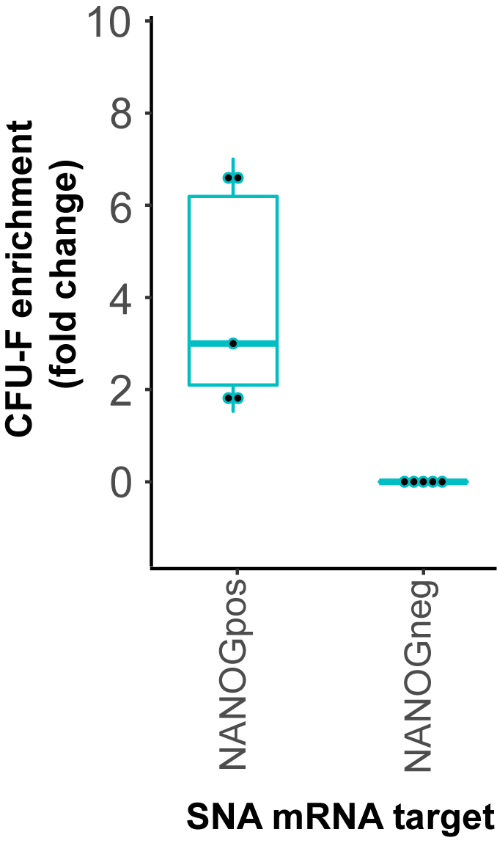


Fig. S5.

**Cells enriched using SNAs targeting NANOG demonstrate enhanced CFU-F capacity in comparison to unselected cells**. SNAs were designed to target a wide range of molecular markers, identified from scRNA-Seq libraries and related literature. The positive and negative populations were plated at 5,000 cells per well of a 12-well plate and colonies were counted after two weeks of culture. Each point represents the mean CFU-F count from a different patient plated as triplicates, displayed as a percentage of unsorted CFU-F counts.


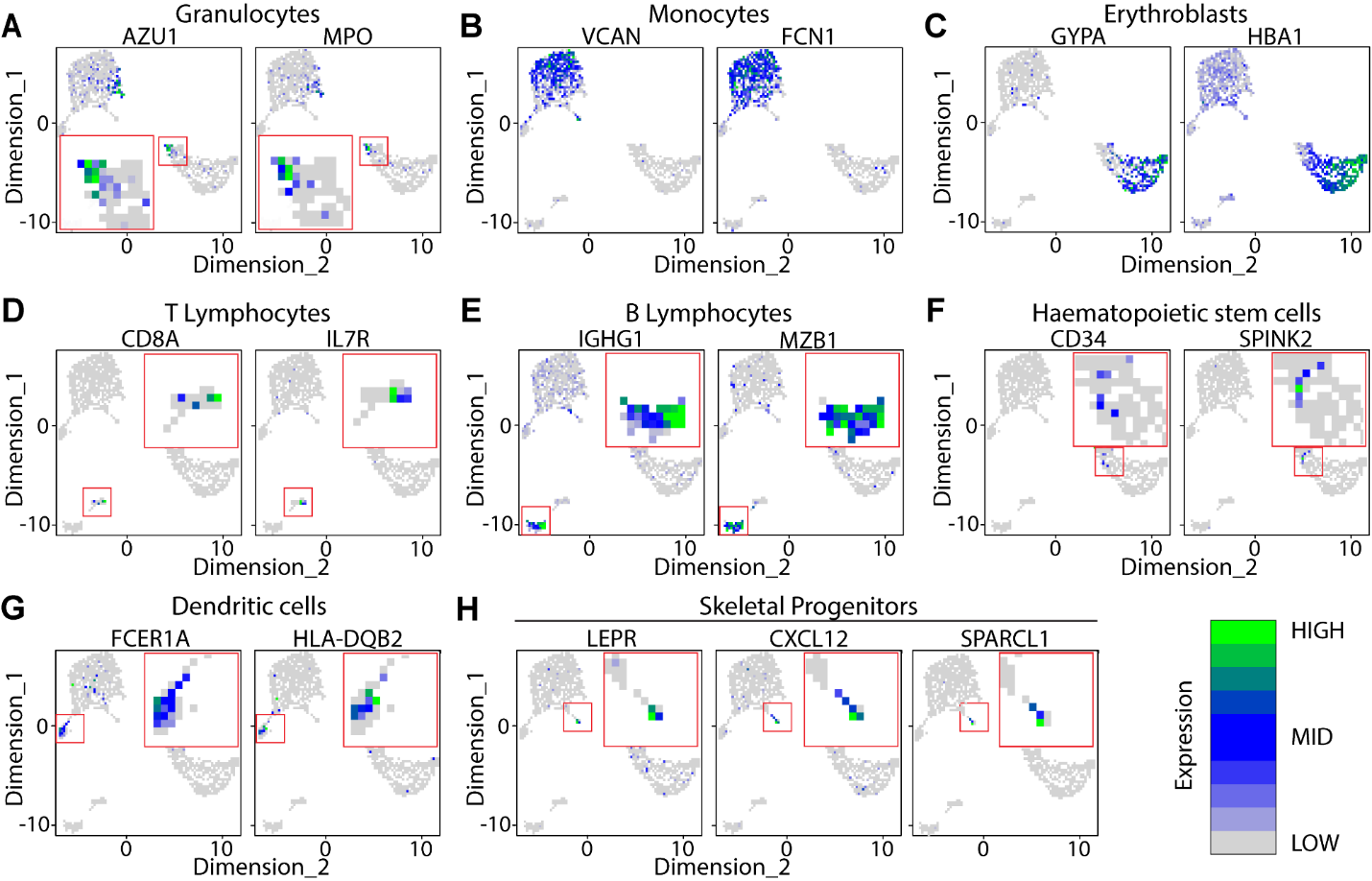


Fig. S6.

Use of lineage biomarkers to characterise Drop-seq data clusters from Stro-1+ cells, and cells enriched using SNAs targeting *CD200, SOST, SPARCL1* and *CD146* mRNAs into 8 cell types; **A)** Granulocytes, **B)** Monocytes, **C)** Erythroblasts, **D)** T Lymphocytes, **E)** B Lymphocytes, **F)** Haematopoietic stem cells, **G)** Dendritic cells**, H)** Skeletal progenitors**.**


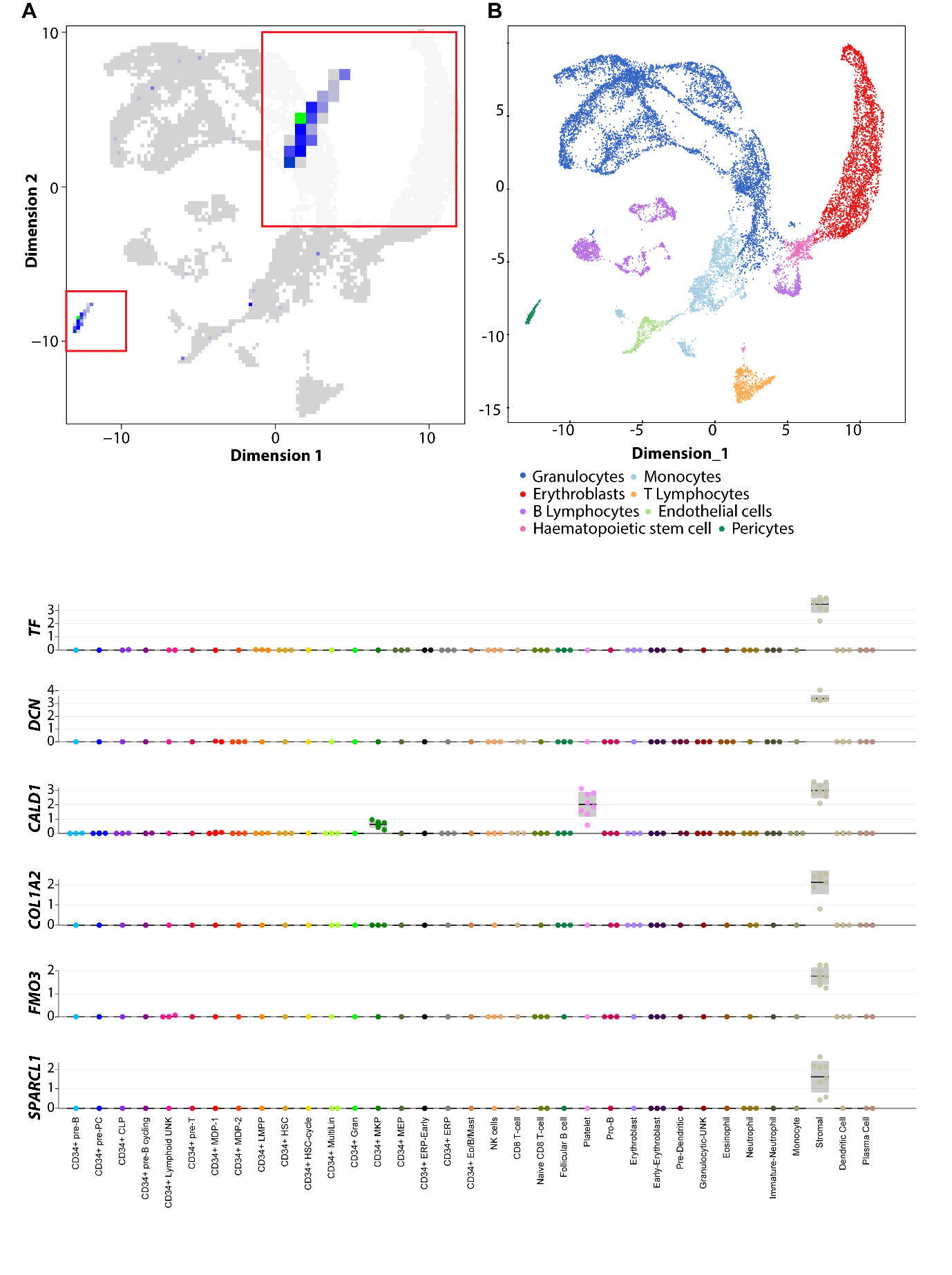


Fig. S7. Expression of *Transferrin (TF)* is elevated in pericyte cell populations.

Visualisation of *TF* expression in the initial scRNA-seq dataset (Drop-seq1) shows elevated expression in the pericyte cluster in comparison to all other cell types. **B)** UMAP of Drop-seq1 annotated with cell subpopulations.


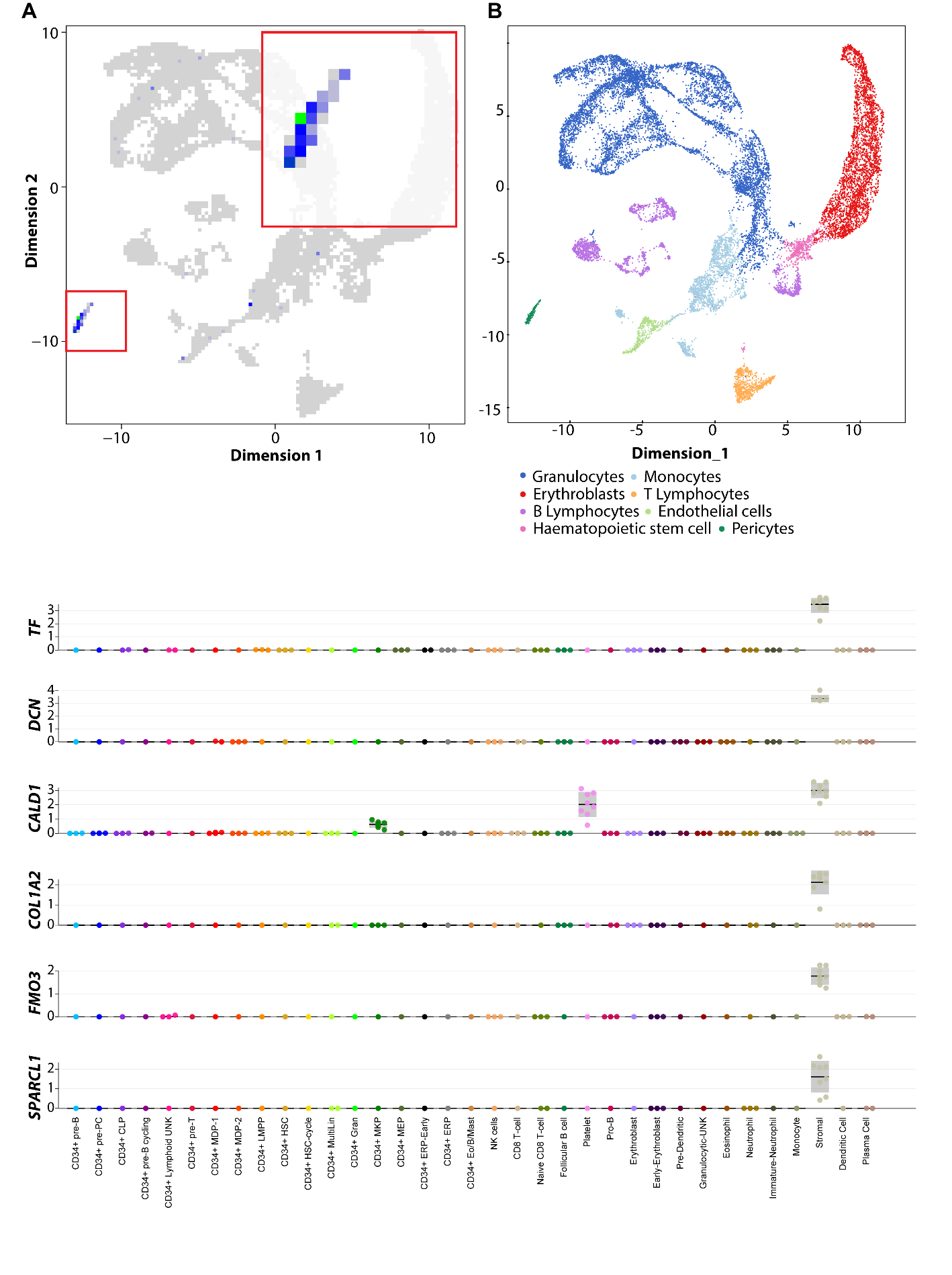


Fig. S8. Expression of key markers identified by scRNA-seq was visualized using the Human Cell Atlas (HCA) bone marrow interactive web portal (1). The HCA bone marrow data comprises over 100,000 cells (haematopoietic and non-haematopoietic) collected from 8 human individual donors. Exploration of the HCA bone marrow portal confirms *TF, DCN, COL1A2, FMO3* and *SPARCL1* characterise cells of stromal identity within human bone marrow. *CALD1* expression is elevated in stromal cells but is also observed in CD34+ Megakaryocyte-erythroid progenitors (MKPs) and platelets.


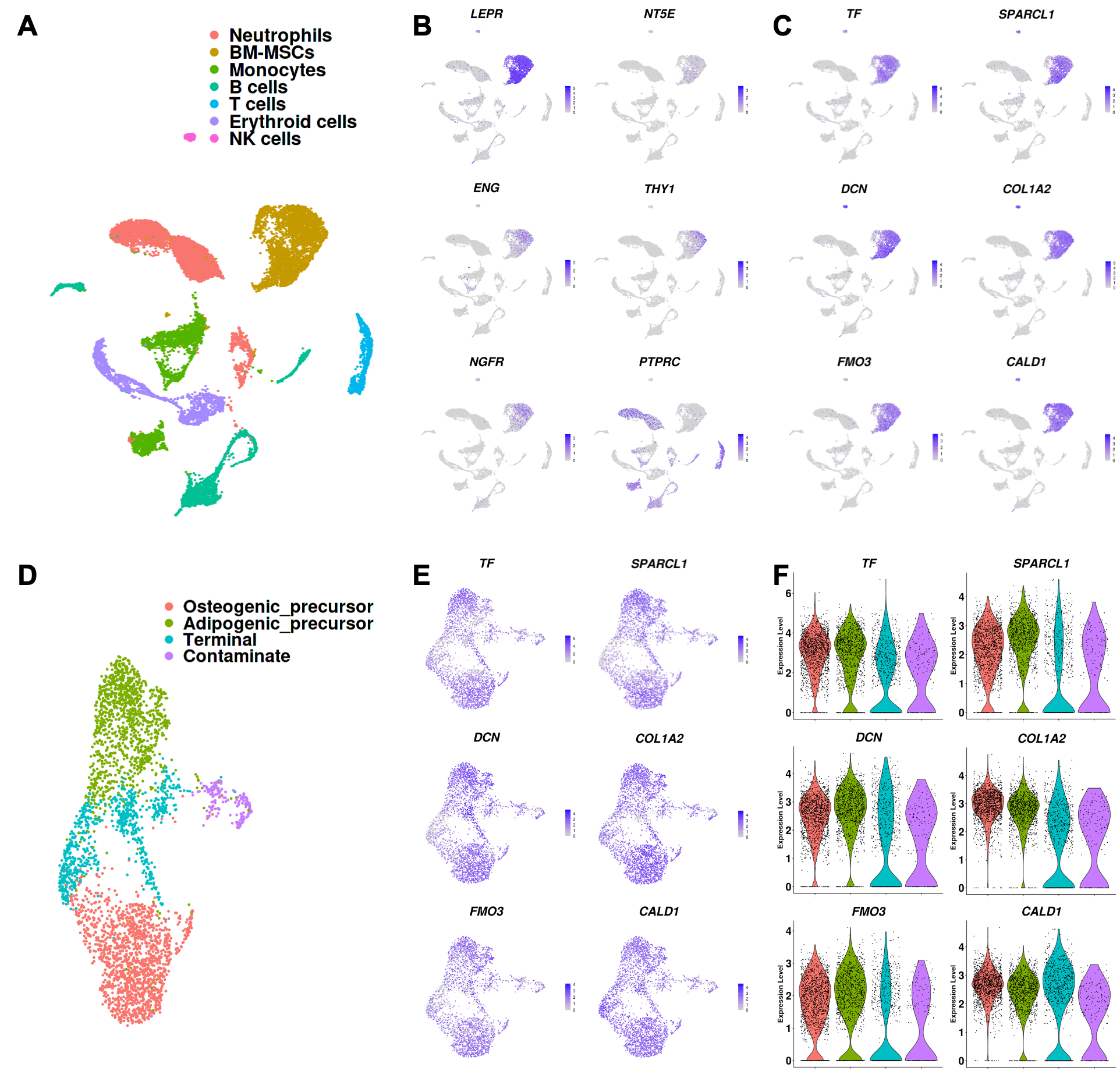


Fig. S9. Analysis of Wang et al scRNA-seq data, profiling CD271^+^ bone marrow mononuclear cells (BM-MNCs) obtained from 2 individual donors. A) The CD271^+^ BM-MNCs represent a heterogenous population. B) Identification of a cluster of *LEPR*^high^*CD45*^low^ cells, expressing SSC signatures. C) Heatmaps visualising expression of candidate SSC targets across CD271^+^ BM-MNCs. D) The *LEPR*^high^*CD45*^low^ cluster can be further subclustered into osteogenic precursors, adipogenic precursors, terminal state cells and contaminate cell populations. E) Heatmaps visualising expression of candidate SSC targets across *LEPR*^high^*CD45*^low^ cells. F) Violin plots show expression of candidate SSC targets across all *LEPR*^high^*CD45*^low^ subpopulations.


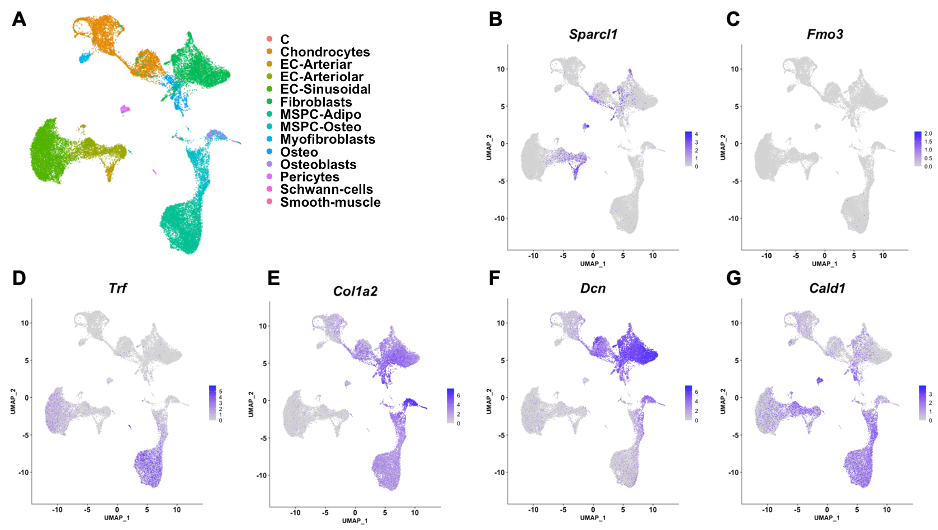


Fig. S10. Expression of key markers identified by scRNA-seq was mapped onto the mouse bone marrow niche scRNA-seq data. The dataset comprised 5 integrated mouse bone marrow niched datasets (>32,000 cells). A) Annotation of cell subpopulations within the data. Heatmap plots were used to visualise expression of mouse orthologs of candidate SSC markers; *Sparcl1,* C) *Fmo3,* D) *Trf,* E) *Col1a2*, F) *Dcn* and G) *Cald1.*


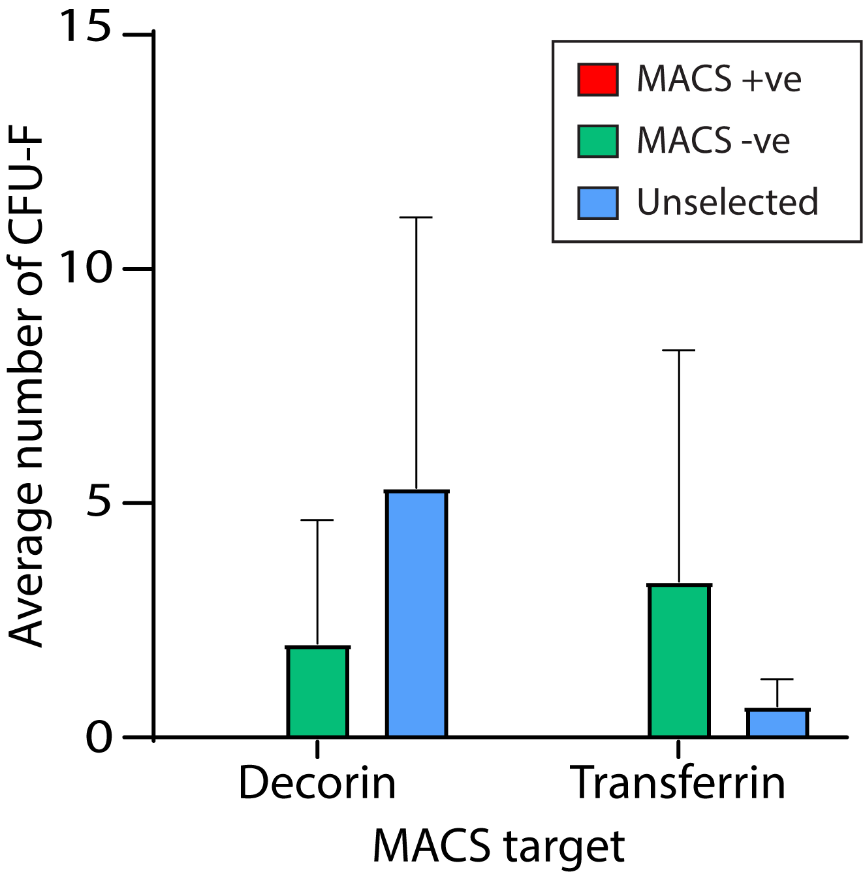


Fig. S11.

Assessment of DCN and TF as targets for SSC enrichment using MACS. MACS was performed to sort HBMSCs based on expression of DCN and TF. Positive and negative fractions, together with unsorted HBMSCs, were plated. After two weeks in vitro, colonies were stained with crystal violet and counted. Data is presented as average CFU-F count.


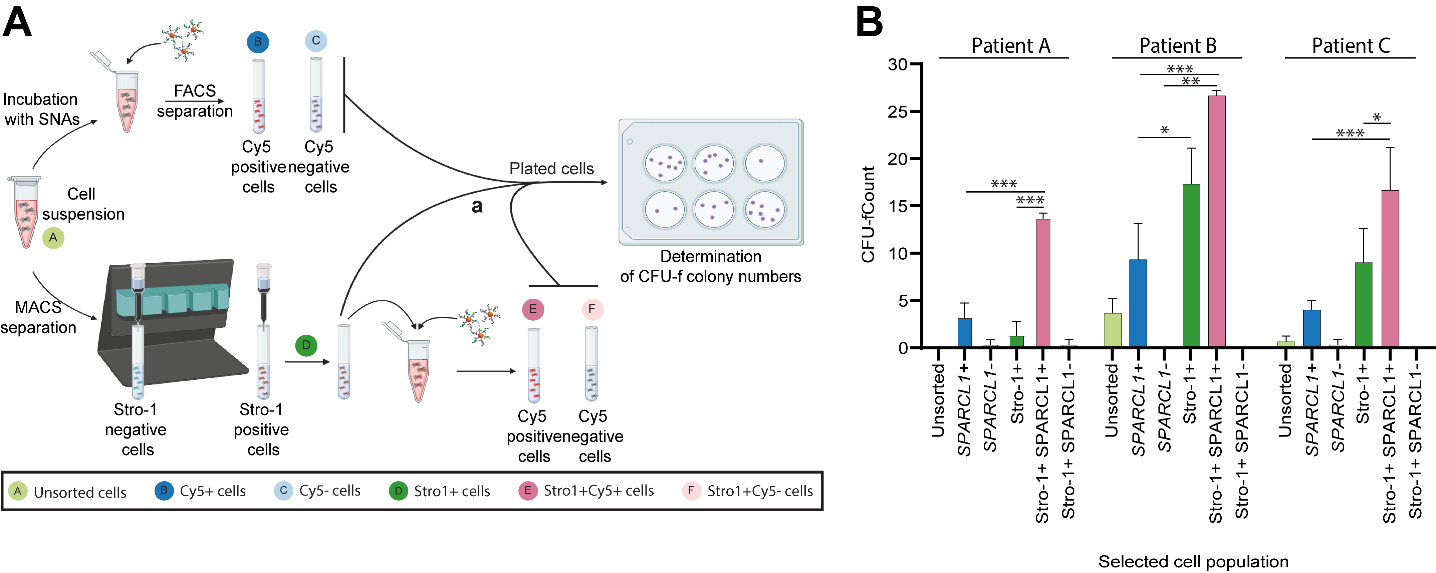


Fig. S12.

**Comparison of *SPARCL1* SNA to Stro-1 antibody for CFU-F isolation. A**). Schematic of experimental protocol for different cell populations collected. **B)** BMMNCs were divided into two parts and processed separately. The first fraction was incubated with SNAs targeting *SPARCL1* mRNA and cells were collected using FACS. Meanwhile, the remainder of the sample was incubated with Stro-1 antibody and Stro-1+ cells were collected using MACS methodology. The Stro-1+ cells were sequentially sorted using the *SPARCL1* SNA to collect Stro1+*SPARCL1+* population. Positive and negative fractions were plated to determine CFU-F enrichment (Figure 3A). Only selected statistics are shown for clarity. * p<0.05, *** p<0.001. Bars show mean with 95% confidence limits. n=3 for each time point for each patient.


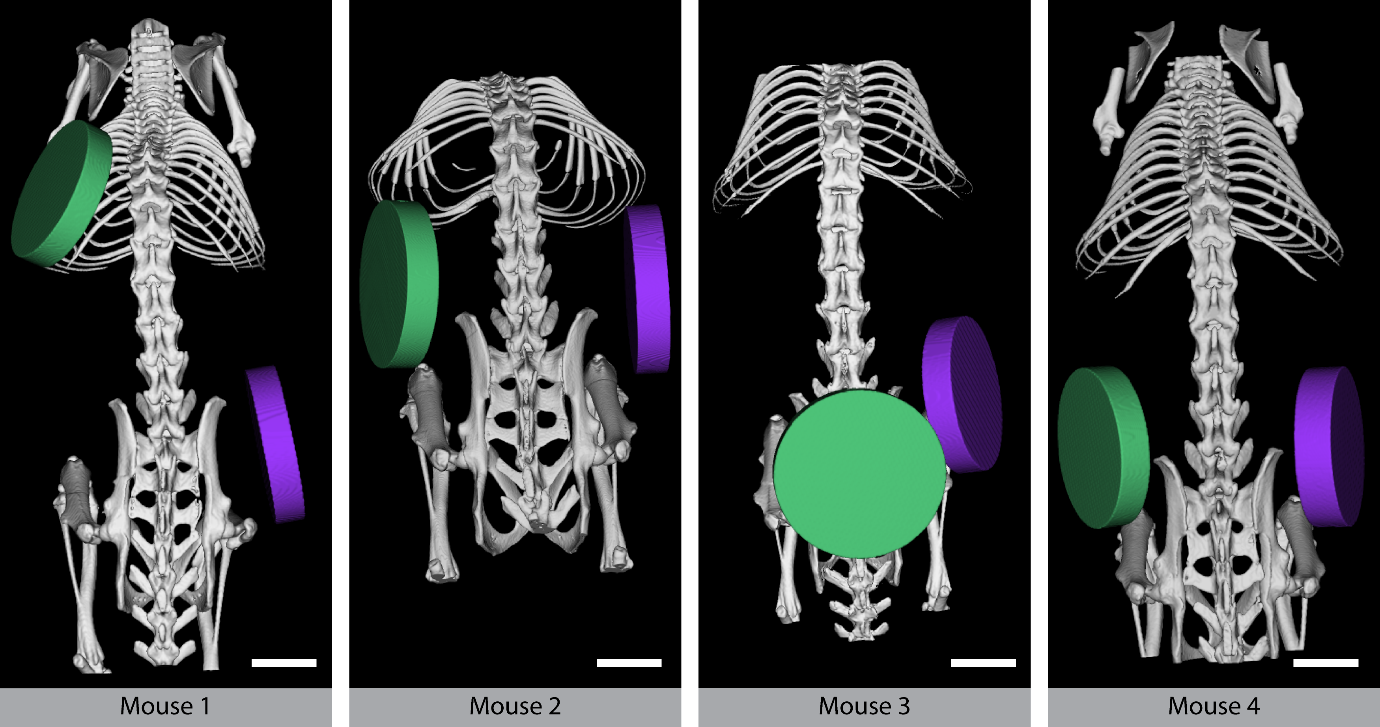


Fig. S13.

Whole body micro-CT scans of mice. Diffusion chambers containing *TF*+ cell-laden alginate/chitosan polysaccharide capsules (green) or acellular capsules (purple) were implanted subcutaneously. Scale bars = 5mm.

Table S1.

Table S1: Sense and flare oligonucleotide sequences. X: 3'-Thiol-Modifier C3 S-S from Link Technologies Ltd.

| SNA Target | Sense Strand Sequence (5’to 3’) with modifications | Flare Strand Sequence (5’ to 3’) with modifications |
| --- | --- | --- |
| *CALD1* | FAM – TATTGCTGCTTGATGGGTCGATAAAAAAAA – X | Cy5 – ATCGACCCATCAAGC |
| *CD73* | FAM – GTTGCGTTCATCAATGGGCGACAAAAAAAA – X | Cy5 – GTCGCCCATTGA |
| *CD146* | FAM – AAGTTCGCTCTTACGAGACGGGAAAAAAAA – X | Cy5 – CCCGTCTCGTAA |
| *CD164* | FAM – GTCTCATGCCTGCGATAGGGACAAAAAAAA – X | Cy5 – GTCCCTATCGCA |
| *CD200* | FAM – GGTCCTGATTCCGGTGACGTTTAAAAAAAA – X | Cy5 – AAACGTCACCGG |
| *Ceruloplasmin* | FAM – TATATTCCATGTACATCGGCCTAAAAAAAA – X | Cy5 – AGGCCGATGTAC |
| *Col1a2* | FAM – CCTCCAACTTAGCCGAAACCTCAAAAAAAA – X | Cy5 – GAGGTTTCGGCTAAG |
| *CXCL12* | FAM – GAATTTTGAGATGCTTGACGTTAAAAAAAA – X | Cy5 – AACGTCAAGCAT |
| *Decorin* | FAM – GCACTTTGTCCAGACCCAAATCAAAAAAAA – X | Cy5 – GATTTGGGTCTGG |
| *FMO3* | FAM – GGTTCCAAATCGAGTGACGAGCAAAAAAAA – X | Cy5 – GCTCGTCACTCGAT |
| *HSPA8* | FAM – AGCAGTACGGAGGCGTCTTACAAAAAAAAA – X | Cy5 – TGTAAGACGCCTC |
| *Nanog* | FAM – ACCATTGCTATTCTTCGGCCAGAAAAAAAAA – X | Cy5 – CTGGCCGAAGAATAG |
| *Osterix* | FAM – ACTCAGGCTCCGGTCCTACAGTAAAAAAAA – X | Cy5 – ACTGTAGGACCG |
| *Podoplanin* | FAM – ACCAATGAAGCCGATGGCTAGTAAAAAAAA – X | Cy5 – ACTAGCCATCGG |
| *RUNX2* | FAM – TGTGGTTGTTTGTGAGGCGAATAAAAAAAA – X | Cy5 – ATTCGCCTCACA |
| *SOST* | FAM – TCTCCGAGCTCGGGGATGATTTAAAAAAAA – X | Cy5 or JOE –  AAATCATCCCCGAGC |
| Scrambled | FAM – ATGGTATACCGAAAGACTGTTAAAAA – X | Cy5 – AACAGTCTTTCG |
| *SPARCL1* | FAM – GTTGGCACCAGAGAGTATTTCAAAAAAAAA – X | Cy5 or JOE –  TGAAATACTCTCTGG |
| *Transferrin* | FAM – GACGCTTTTCATATGGTCGCGGAAAAAAAA – X | Cy5 – CCGCGACCATATG |

Supplementary references.

1. Hay SB, Ferchen K, Chetal K, Grimes HL, Salomonis N. The Human Cell Atlas bone marrow single-cell interactive web portal. *Experimental Hematology*. 2018;68:51-61.
